# SERS characterization of colorectal cancer cell surface markers upon anti‐EGFR treatment

**DOI:** 10.1002/EXP.20210176

**Published:** 2022-05-09

**Authors:** Nana Lyu, Bernadette Pedersen, Elena Shklovskaya, Helen Rizos, Mark P. Molloy, Yuling Wang

**Affiliations:** ^1^ ARC Center of Excellence for Nanoscale BioPhotonics and School of Natural Sciences, Faculty of Science and Engineering Macquarie University Sydney New South Wales Australia; ^2^ Department of Biomedical Sciences, Faculty of Medicine, Health and Human Sciences Macquarie University Sydney New South Wales Australia; ^3^ Bowel Cancer and Biomarker Laboratory, School of Medical Sciences, Kolling Institute The University of Sydney Sydney New South Wales Australia

**Keywords:** anti‐EGFR treatment, colorectal cancer, epidermal growth factor receptor, phenotypic evolution, surface‐enhanced Raman spectroscopy (SERS)

## Abstract

Colorectal cancer (CRC) is the third most diagnosed and the second lethal cancer worldwide. Approximately 30–50% of CRC are driven by mutations in the *KRAS* oncogene, which is a strong negative predictor for response to anti‐epidermal growth factor receptor (anti‐EGFR) therapy. Examining the phenotype of *KRAS* mutant and wild‐type (WT) CRC cells in response to anti‐EGFR treatment may provide significant insights into drug response and resistance. Herein, surface‐enhanced Raman spectroscopy (SERS) assay was applied to phenotype four cell surface proteins (EpCAM, EGFR, HER2, HER3) in *KRAS* mutant (SW480) and WT (SW48) cells over a 24‐day time course of anti‐EGFR treatment with cetuximab. Cell phenotypes were obtained using Raman reporter‐coated and antibody‐conjugated gold nanoparticles (SERS nanotags), where a characteristic Raman spectrum was generated upon single laser excitation, reflecting the presence of the targeted surface marker proteins. Compared to the *KRAS* mutant cells, *KRAS* WT cells were more sensitive to anti‐EGFR treatment and displayed a significant decrease in HER2 and HER3 expression. The SERS results were validated with flow cytometry, confirming the SERS assay is promising as an alternative method for multiplexed characterization of cell surface biomarkers using a single laser excitation system.

## Graphical abstract

Examining the phenotypes of KRAS mutant and wild‐type colorectal cancer cells during anti‐EGFR treatment may provide significant insights into drug resistance. Herein, we applied surface‐enhanced Raman spectroscopy (SERS) assay to detect four cell surface proteins on KRAS mutant and wild‐type cells over a 24‐day treatment, where SERS assay achieved multiplexed characterization of cell surface markers using a single laser excitation.

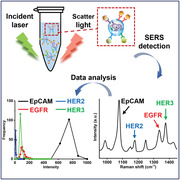

## 1 INTRODUCTION

Colorectal cancer (CRC) is the third most diagnosed and the second most lethal cancer.^[^ ^1^ ^]^ It has been reported that up to 50% of patients will develop metastases during their disease course,^[^ ^2^ ^]^ and the 5‐year survival from patients diagnosed with advanced CRC is only 13% (data from Australia Institute of Health and Welfare 2019). Targeted therapy approaches have focussed on the epidermal growth factor receptor (EGFR), which is overexpressed in approximately 80% of CRC.^[^ ^3^ ^]^ Activation of EGFR upon ligand binding and its subsequent downstream signalling pathways will promote cell growth and proliferation.^[^ ^3c^ ^]^ The emergence of cetuximab and panitumumab, which are two anti‐EGFR monoclonal antibodies, marks an era toward the personalized therapy for metastatic CRC (mCRC).^[^ ^3^, ^4^ ^]^ However, the overexpression of EGFR does not always indicate a positive answer to anti‐EGFR therapy.^[^ ^3b^ ^]^ The downstream signalling effector, *KRAS*, is the mediator for linking the binding of extracellular ligand with the intracellular signal transduction from EGFR to the nucleus. Thus, the activation of *KRAS* mutation is used as a negative indicator for a lack of answer to anti‐EGFR treating.^[^ ^3^, ^4^ ^]^ While WT *KRAS* tumours are indicated for adoption of anti‐EGFR treatment, not all patients will respond,^[^ ^5^ ^]^ so predictive features are required for insights into the response and resistance to EGFR‐targeted therapy in CRC.

The innate (primary) and acquired mechanisms of EGFR inhibitor resistance have been proposed.^[^ ^6^ ^]^ Innate resistance refers to the refractoriness to initial therapy and could be explained by pre‐existing resistance mechanisms, such as activating mutations in downstream EGFR effectors, including *KRAS*, *BRAF* and *PIK3CA* mutations or *PTEN* loss. These alterations cause continuous activation of the downstream signalling despite inhibition of EGFR. Acquired resistance is defined as development of treatment resistance after an initial response to therapy.^[^ ^7^ ^]^ Nearly all patients with mCRC who respond to EGFR‐targeted therapy at beginning, will finally progress on treatment.^[^ ^4^, ^6^ ^]^ The acquired resistance to EGFR‐targeted treatment includes acquired alterations that directly activate EGFR signalling (e.g. *EGFR* mutations, *KRAS/BRAF* alterations) or activation of alternate signalling pathways (e.g. activation of epidermal growth factor receptor‐2 (HER2) and mesenchymal‐epithelial transition factor receptor (MET)).^[^ ^4^, ^7^, ^8^ ^]^ Therefore, monitoring phenotypic features of cancer cells during therapy will provide new insights on cancer cells treatment response and resistance.

The strategies for phenotyping the cells involve protein‐based methods for instance flow cytometry, while commonly used, a large number of cells are required and the capabilities for multiplexed detection are limited due to the overlapping of fluorescence signals.^[^ ^9^ ^]^ Though Western blotting is one of the most common procedures for protein detection, it is applied to detect specific protein molecules from a variety of proteins associated with a particular tissue or cell type, where the target protein was detected by probing with antibodies after separation of the proteins by gel electrophoresis and membrane blotting.^[^ ^10^ ^]^ Cytometry by time of flight (CyTOF) as an emerging approach may overcome some of these limitations, but does not allow analysis of live cells, the equipment is not widely available, and the cost of metal conjugated antibodies and antibody conjunction kits are expensive.^[^ ^11^ ^]^ Other techniques such as real‐time quantitative reverse transcription PCR (qRT‐PCR) which is based on the analysis of nucleic acid, are able to detect the relative expression of epithelial or tumour specific mRNAs, it is unable to directly characterize multiple cell surface markers and determine their heterogeneity.^[^ ^9^, ^12^ ^]^ More recently, single‐cell RNA sequencing (scRNA‐seq) has become the choice method for investigating key biological questions of cell heterogeneity, but this technology is expensive and requires significant expertise.^[^ ^13^ ^]^ Thus, an innovative assay allowing direct phenotypic characterization of multiple surface biomarkers on the same cells in a single sample is highly desired.

Herein, we describe a strategy for monitoring the phenotypic evolution of *KRAS* mutant and WT CRC cells during anti‐EGFR treatment by simultaneous detection of multiple cell surface markers by surface‐enhanced Raman spectroscopy (SERS), which is based on a Raman spectroscopic assay to detect molecules in close proximity to the nanostructure surface, the characteristic Raman spectra resulting from localized surface plasmon resonance endows detection of the target molecules with ultra‐sensitivity.^[^ ^14^ ^]^ In recent years, SERS assay has demonstrated core superiorities including ultra‐sensitivity for probing single molecule,^[^ ^15^ ^]^ multiplexing capabilities due to the sharp Raman bands, peak intensity based quantification, high photostability, minimal auto‐fluorescence from biological samples under a single laser excitation with red to near‐infrared laser wavelength.^[^ ^14^, ^16^ ^]^ Therefore, compared to the conventional flow cytometry, SERS assay offers the key advantages including (i) simple and affordable single laser system for characterizing multiple cell surface proteins expression in one measurement, while flow cytometry requires different and multiple laser excitation wavelength for different fluorophores; (ii) high sensitivity as only 100–1000 cells are required for SERS analysis; (iii) highly multiplexed capability of SERS nanotags owing to the narrow peaks of Raman spectrum, while spectral overlapping with flow cytometry fluorophores requires compensation. The main disadvantage of SERS assay lies on the preparation of SERS nanotags, which requires the monodisperse and stable nanoparticles to ensure the reproducibility of the measurement, this limitation can be overcome by adopting the automated nanoparticle synthesis system.^[^ ^17^ ^]^

Herein, the aim of this report is to propose the application of SERS assay in analysing the responses of multiple markers on cell surface during treatment, by fully taking the advantages of SERS assay. To study the phenotypic evolution of *KRAS* mutant cells (SW480) and *KRAS* WT cells (SW48), we selected four cell surface markers, which involve 1) epithelial cell adhesion molecule (EpCAM) with expression in 85% of colorectal carcinomas;^[^ ^18^ ^]^ 2) EGFR, a member of EGFR family, overexpressed in approximately 80% of CRC;^[^ ^3b,c^ ^]^ 3) HER2, a member of EGFR family whose overexpression was potentially predictive of anti‐EGFR resistance in CRC;^[^ ^19^ ^]^ and 4) HER3, another EGFR family member expressed in 70% of primary CRC, and the high expression of HER3 is correlated with worse outcomes in clinic.^[^ ^20^ ^]^ The specific antibodies for targeting the respective markers were conjugated to SERS nanotags (AuNPs coated with Raman reporter), where the fingerprint of Raman spectrum for each SERS nanotag was generated upon excitation with only a single laser wavelength, indicating the presence of SERS nanotags and reflecting the existence of the targeting surface marker. The SERS assay was thus used for monitoring the phenotypic evolution of these marker proteins during cetuximab treatments, and the results were validated by a standard technique, namely flow cytometry. The SERS assay and flow cytometry delivered comparable results demonstrating that SW48 cells were more sensitive to EGFR‐targeted therapy with significant downregulation of HER2 and HER3 expression levels compared to SW480 cells. The expression of EpCAM and EGFR in both SW48 and SW480 remained stable during EGFR‐targeted therapy.

## 2 RESULTS AND DISCUSSION

### 2.1 Working scheme

**FIGURE 1:**
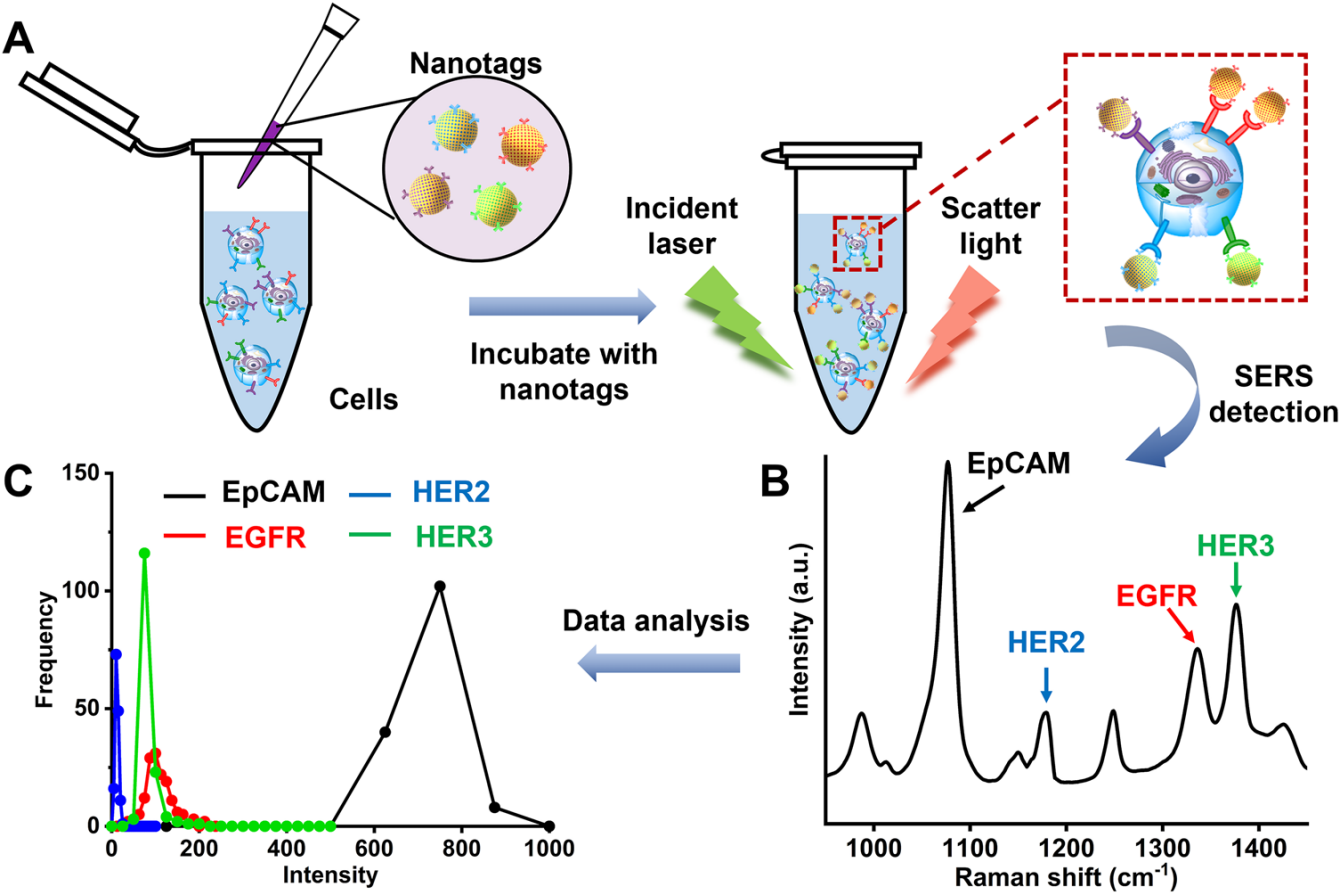
(A) Schematic illustration of cell surface markers labelling using antibody‐conjugated SERS nanotags for targeting EpCAM, EGFR, HER2 and HER3. (B) Averaged SERS spectrum for the bulk ensemble of the labelled cells in the scattering volume. (C) The expression level distribution of surface markers for each sample plotted by the frequency and the Raman signal intensity with the characteristic peaks at 1078, 1180, 1340 and 1380 cm^−1^ for EpCAM, HER2, EGFR and HER3, respectively

Figure 1 illustrates the working scheme of our proposed SERS approach for phenotypic characterization of four cell surface markers of CRC cells. Briefly, the cells were collected and resuspended in PBS, followed by incubating with the four different Ab‐SERS nanotags which were functionalized with specific Raman reporters and antibodies for targeting EpCAM, EGFR, HER2 and HER3 (Figure 1A) to allow the simultaneous detection of cell surface markers by Raman spectroscopy.^[^ ^9^ ^]^ Given that the distinct peaks of the SERS nanotags are easily identified, the multivariate statistical methods are thus not required in this analysis.^[^ ^21^ ^]^ The averaged SERS spectrum (Figure 1B) represents the bulk overall of the cells with labelling for Raman scattering, in which the intensity of Raman signal reflects the relative expression levels of the four cell surface markers. SERS signal intensity at the characteristic peak was read from the y‐axis (Intensity) of Raman spectrum for the corresponding SERS nanotags (Figure 1B). The SERS signal intensities of the characteristic peaks are specific for the respective biomarkers including 1078, 1180, 1340 and 1380 cm^−1^ for EpCAM, HER2, EGFR and HER3, respectively.^[^ ^9^, ^22^ ^]^ The cell surface markers expression distribution (Figure 1C) of each sample was analysed by plotting the frequency and Raman signal intensity of the characteristic peaks (Figure 1B) at 1078 cm^−1^ (EpCAM), 1180 cm^−1^ (HER2), 1340 cm^−1^ (EGFR) and 1380 cm^−1^ (HER3), respectively.

### 2.2 Synthesis and characterization of SERS nanotags

**FIGURE 2:**
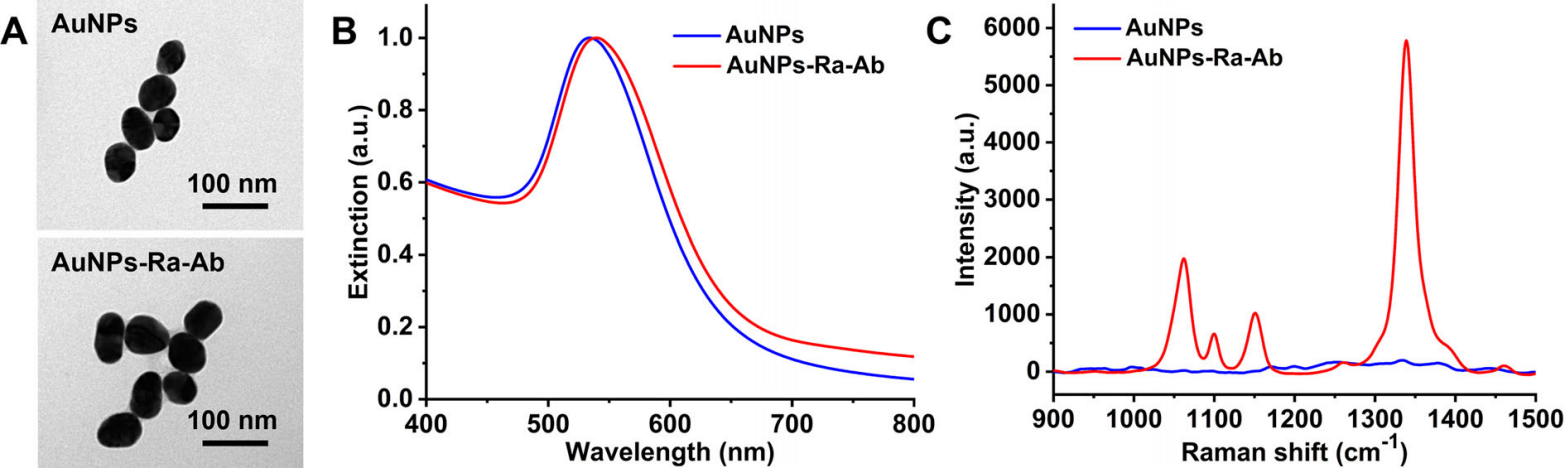
Characterization of gold nanoparticles (AuNPs) and AuNPs‐Ra‐Ab. TEM images (A), normalized UV–vis spectra (B) and SERS spectra (C) of AuNPs before and after coating with Raman molecule (Ra, DTNB)) and anti‐EGFR antibody

Our previous study has demonstrated that higher SERS activity could be obtained by using AuNPs (60 nm) as compared to smaller AuNPs (i.e., 25 and 40 nm).^[^ ^23^ ^]^ Meanwhile, 60 nm AuNPs are less prone to aggregation compared to larger AuNPs at size of 80 nm.^[^ ^23^ ^]^ Thus, AuNPs (60 nm) were used in this study. The size and morphology of AuNPs were investigated with transmission electron microscopy (TEM), where the quasi‐spherical shape of as‐prepared AuNPs was observed (Figure 2A). UV–vis absorption spectroscopy was tested to characterize the surface plasmon resonance of AuNPs (60 nm) (Figure 2B), in which the wavelength of maximum absorption for AuNPs shows red shift from 535 to 539 nm after adsorption of Raman molecules (Ra, DTNB) and conjugation with antibody (Ab, anti‐EGFR antibody).

Dynamic light scattering (DLS) was adopted for analysing AuNPs size distribution with and without functionalization with Ra and Ab (AuNPs‐Ra‐Ab) (Figure S1), which shows the size increases from 68.1 to 78.8 nm, demonstrating the binding of Ra and Ab on the surface of AuNPs. Electrophoretic light scattering (ELS) was used to determine the zeta potential of AuNPs and AuNPs‐Ra‐Ab, where the AuNPs with citrate‐capping show negative charge with zeta potential of −23.4 mV (Table S1). AuNPs became less negative with the change of zeta potential from −23.4 to −20.1 mV after conjugating with anti‐EGFR antibody (isoelectric point 8.5), which was positively charged in PBS of pH 7.4.^[^ ^23^, ^24^ ^]^ The Raman spectrum of a typical SERS nanotag (coated by DTNB and Ab) displayed distinct signal enhancement effect for DTNB (Figure 2C), which is attributed to the electromagnetic effect induced by localized surface plasmon resonance.^[^ ^21^, ^25^ ^]^

### 2.3 Cell surface biomarkers characterization before drug treatment

**FIGURE 3:**
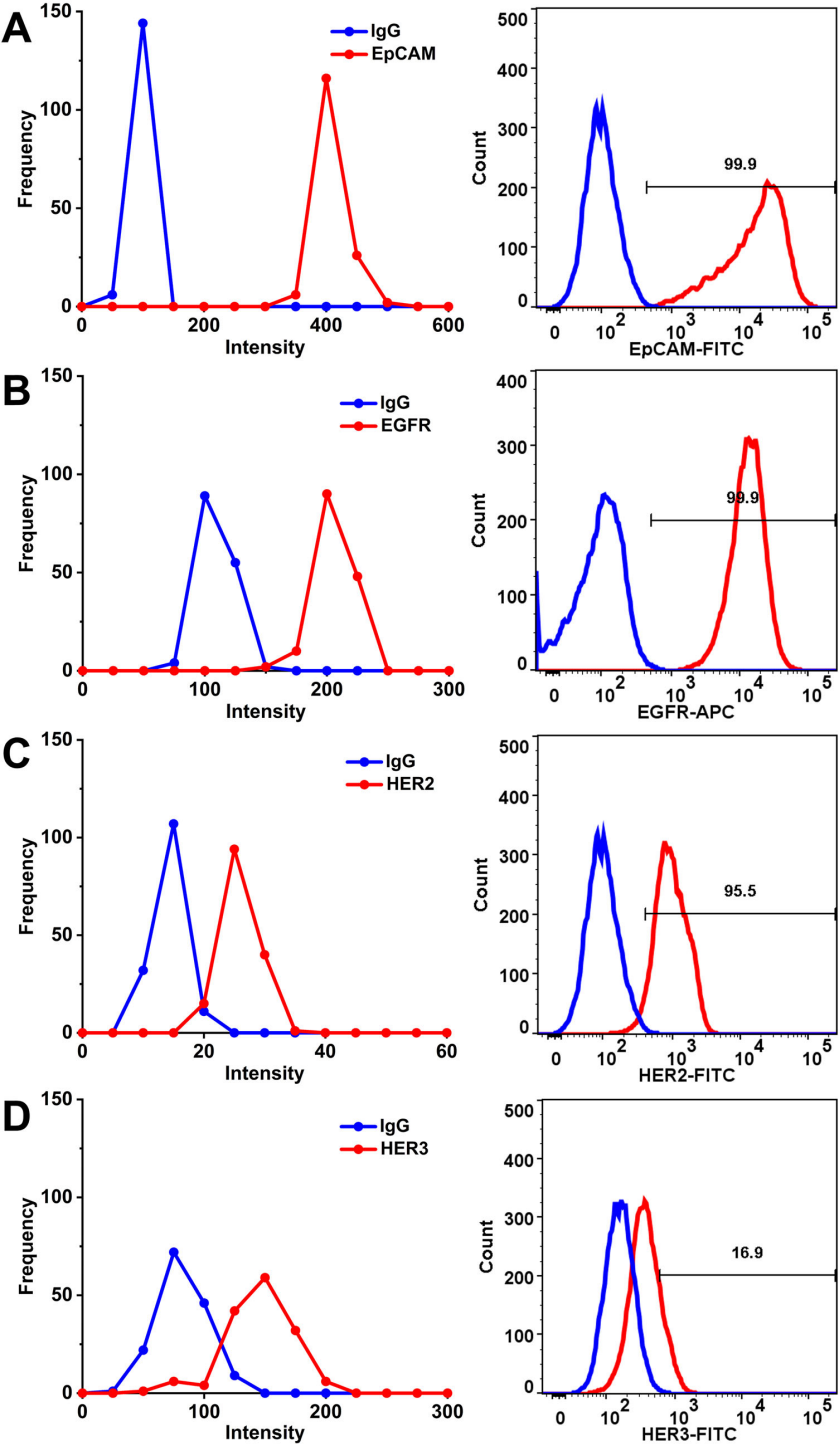
Surface marker expression distribution/profile for SW480 cells before drug treatment. Distribution of four markers (EpCAM, EGFR, HER2 and HER3) on cells’ surface obtained by SERS assay (left) and flow cytometry (right)

SERS technique was used to study the expression levels of four cell surface proteins in *KRAS* mutant (Figure 3, left) and WT cells (Figure S2, left). The narrower expression frequency vs. intensity curve for the respective markers indicated the more homogeneity of phenotypes, which usually resulted from the selection or adaptation of subclones in response to treatment.^[^ ^9^ ^]^ In contrast, the wider frequency vs. intensity distribution reflects the more diverse and heterogeneous cells population.^[^ ^9^ ^]^ The expression level of the respective biomarkers was identified by comparing the overlap of expression distribution curve between the biomarkers and isotype‐matched IgG control. Therefore, SERS assay for SW480 and SW48 cells shows that EpCAM and EGFR have higher expression levels with well‐separated distribution curves compared with IgG control, HER2 curve shows a slightly lower expression exhibiting only a small portion of overlapping with that of IgG control (Figure 3), while HER3 on SW480 shows lower expression exhibiting more overlapping distribution profile with IgG control, compared to SW48 cells (Figure S2, left). The sensitivity of using each Ab‐SERS nanotag alone and in combination for cell detection was explored by titrating 10–1000 cells into 1 ml of PBS, each Ab‐SERS nanotag alone and in combination enable the detection down to 10 cells, demonstrating that the SERS assay is sensitive for cell characterization.^[^ ^9^ ^]^

Flow cytometry (Figure 3, right) was adopted as a conventional assay to verify the results from SERS assay. As shown in Figure 3 (right), EpCAM, EGFR and HER2 illustrated higher expression levels (99.9%, 99.9% and 95.5%, respectively) on SW480 cell surface, while relatively lower expression (16.9%) of HER3 aligns well with SERS distribution profiles (Figure 3, left). It was reported that mRNA expression of HER3 in tumor may be conceived as a useful biomarker for predicting anti‐EGFR therapeutic efficacy in CRC, where the heregulin activation of HER3 may activate an alternate signalling pathway to circumvent EGFR‐inhibition, which supports the overexpression of HER3 as a negative indicator for anti‐EGFR therapy in CRC.^[^ ^26^ ^]^

The cell surface markers EpCAM, EGFR and HER2 of WT SW48 cells were detected with high expression (99.7%, 99.6% and 99.7%, respectively). While HER3 showed slightly higher expression (83.6%) on WT SW48 cells compared to *KRAS* mutant SW480 cells, which is in line with the published reports.^[^ ^20^, ^27^ ^]^ The intensity distribution obtained by SERS detection is comparable to the flow cytometry, demonstrating that both techniques were capable of distinguishing the positive cell markers from the isotype control.

### 2.4 Cell surface protein changes in response to EGFR‐inhibition

We further used SERS assay and flow cytometry to evaluate cell surface expression in SW480 cells following anti‐EGFR (cetuximab) treatment over a 24‐day time course, where the intensity distribution of cell surface markers obtained by SERS assay (Figures S3–S7 left, and Figure S8) is comparable to the results from flow cytometry (Figures S3–S7 right, and Figure S9).

**FIGURE 4:**
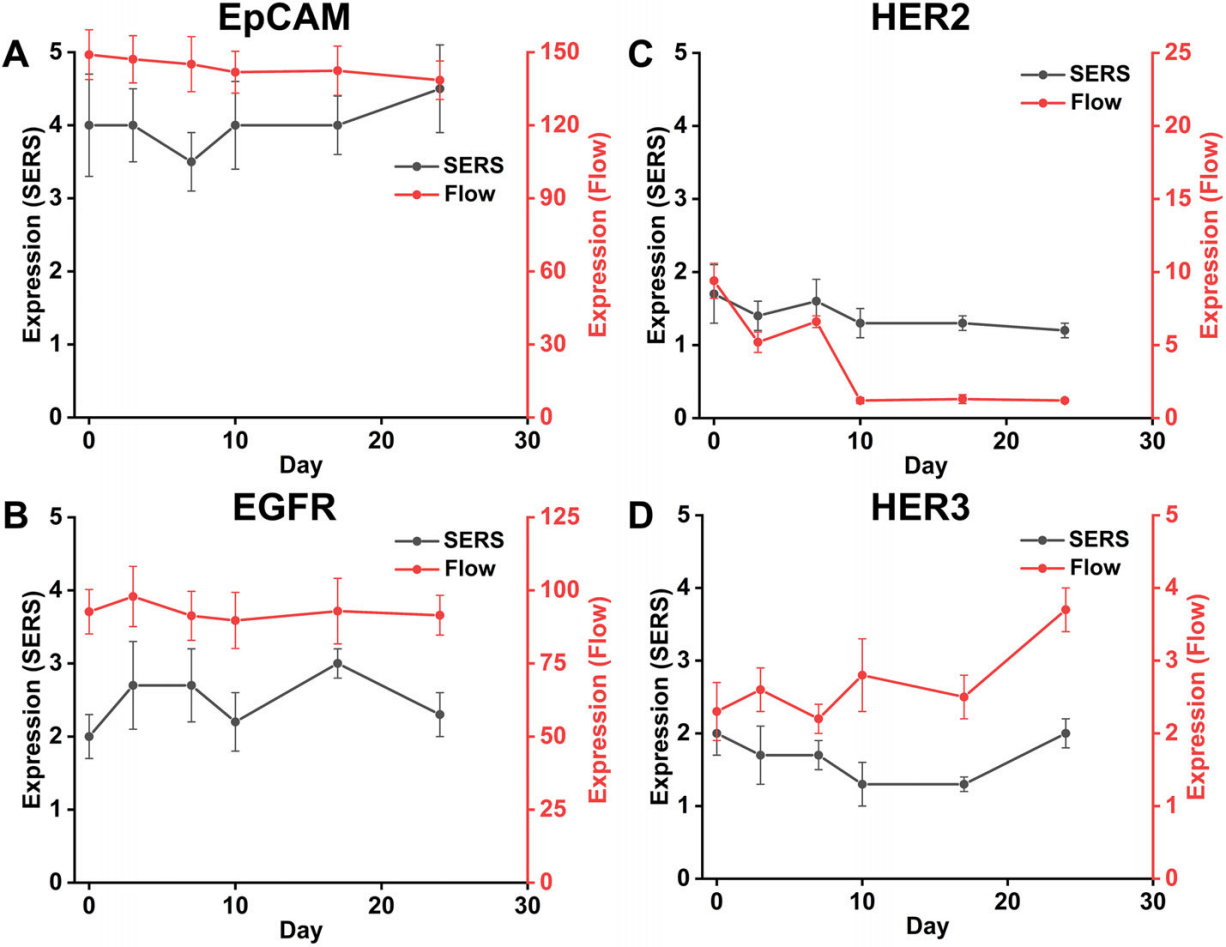
Evolution of surface marker expression profiles for *SW480* cells before drug treatment (0 day) and upon cetuximab treatment for 3, 7, 10, 17 and 24 days. The expression of four markers (EpCAM, EGFR, HER2 and HER3) was presented by the ratio of stained and control samples for median intensity from SERS assay and MFI from flow cytometry

The expression of cell surface markers was further demonstrated by the ratio of stained and control samples for median intensity from SERS assay and median fluorescence intensity (MFI) from flow cytometry, as indicated in Figure 4. The SW480 EpCAM expression detected by SERS assay showed a slight decrease after treatment for 3 days, then it increased with the treatment course (7, 10, 17 and 24 days), as shown in Figure 4A. The expression of EGFR on SW480 cells determined by flow cytometry showed no significant changes upon treatment for 24 days (Figure 4B), while the expression level from SERS assay presented fluctuations during treatment (Figure 4B). Furthermore, the SERS assay demonstrated that EGFR on SW480 cells slightly increased, and signal distribution broadened with longer time of treatment (7, 10 and 17 days, Figure S8B), reflecting the more diverse and heterogeneous cell populations evolving during cetuximab treatment. Therefore, EpCAM and EGFR were characterized with high expression by both SERS assay and flow cytometry, and the more dramatic change of the expression upon treatment detected by SERS (Figure 4A,B) may be ascribed to the ultra‐sensitivity of SERS assay for identifying the subtle variations of phenotypic characters of treated cells.

Decreased expression of HER2 was observed during treatment with cetuximab for 24 days (Figure 4C) compared to cells before treating (0 day), the consistent evolution trend for HER2 expression were obtained from both SERS assay and flow cytometry, where obvious decrease of HER2 expression was observed upon treatment, overall, the expression of HER2 decreased significantly from 95.5% (Figure 3) to 0.26% (Figure S7) after cetuximab treatment for 24 days. HER3 was observed with decreased levels upon cetuximab treatment for 3, 7 and 10 days, then the expression of HER3 increased after longer time of treatment for 17 and 24 days (Figure 4D), the results were also validated with flow cytometry, where the evolution trend of HER3 expression showed fluctuations during treatment. While for HER2 with slightly lower expression and HER3 with lower expression, the evolution upon treatment detected by SERS shows similar pattern with that of flow cytometry.

**FIGURE 5:**
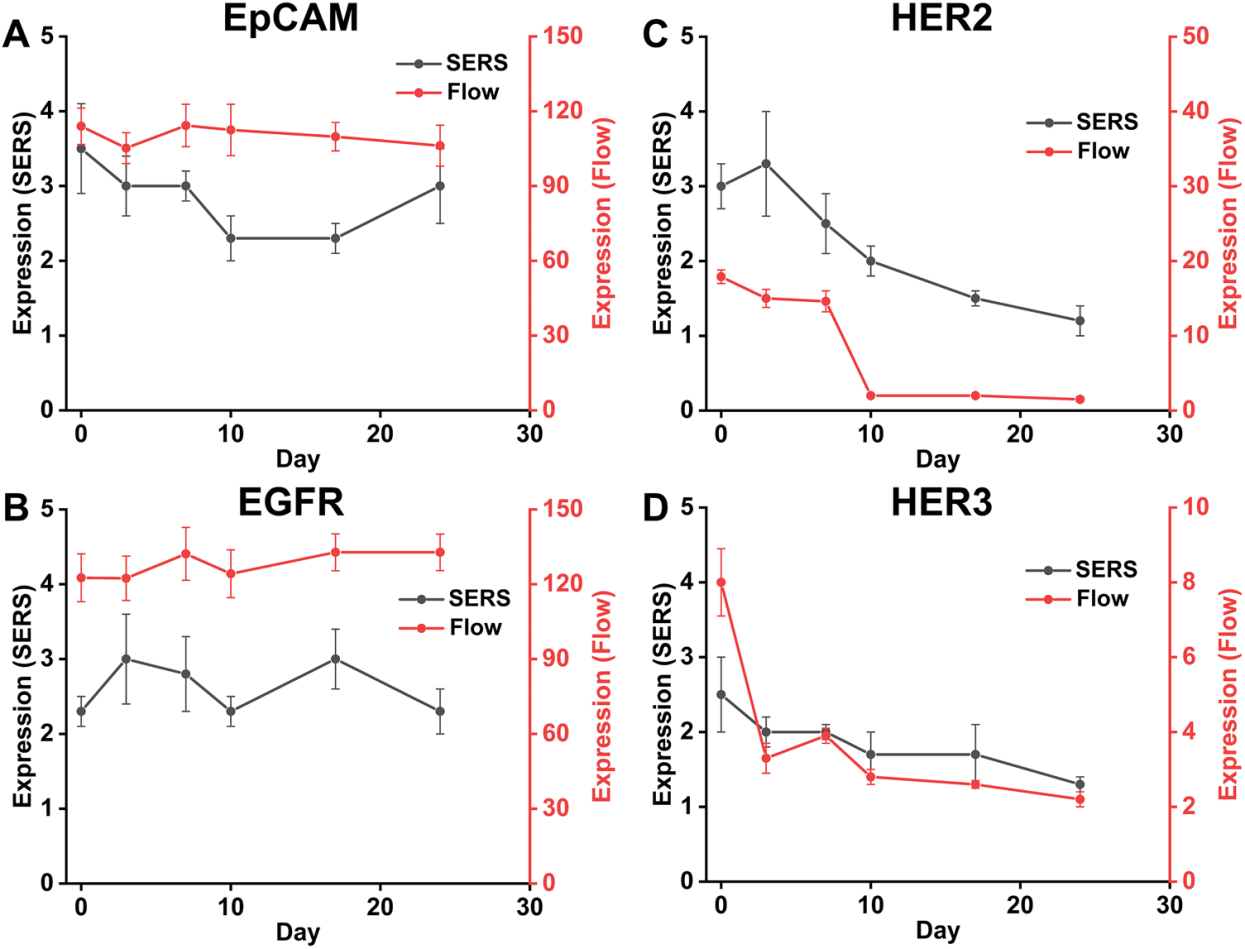
Evolution of surface marker expression profiles for *SW48* cells before drug treatment (0 day) and upon cetuximab treatment for 3, 7, 10, 17 and 24 days. The expression of four makers (EpCAM, EGFR, HER2 and HER3) was also presented by the ratio of stained and control samples for median intensity from SERS detection and MFI from flow cytometry

With regards to WT (SW48) cells, the evolution of four markers (EpCAM, EGFR, HER2 and HER3) expression was monitored before (0 day) and during drug treatment (3, 7, 10, 17 and 24 days), as illustrated in Figures S10–S16. The expression of EpCAM and EGFR showed no significant change during cetuximab treatment for different time points (3, 7, 10, 17 and 24 days) compared to cells without treatment (0 day), as illustrated in SERS assay (Figure S15; Figure 5A,B). The results were also validated with flow cytometry, where the expression of EpCAM and EGFR kept stable with > 99.0% expression over cetuximab treatment for 24 days (Figure S16). While, SERS assay (Figure S15) showed significant decrease of HER2 and HER3. SERS results were also validated with flow cytometry, which showed significant expression decrease of HER2 and HER3 from 99.7% to 0.03% and 83.6% to 0.04%, respectively, during the treatment time of 24 days.

In addition, the expression changes of surface markers over time detected by both SERS assay and flow cytometry were analysed by the ratio of stained and control samples for median intensity from SERS assay and MFI from flow cytometry. Although the median intensity ratio of stained and control samples from these two assays are not identical at all‐time points during treatment, the overall trends of the curves from these two assays during treatment are consistent, the results are also validated by surface maker expression profiles (Figures S2–S7 and S10–S14). As indicated in Figure 5, the downregulation of both HER2 and HER3 has been demonstrated for SW48 cells.

Overall, CRC *KRAS* mutant cells (SW480) and WT cells (SW48) displayed distinct phenotypic evolution after cetuximab exposure for 24 days with respect to the four markers measured. For *KRAS* mutant SW480 cells, expression levels of EpCAM, EGFR showed slight increase within treatment for 17 days, then decreased at 24 days; HER2 and HER3 expression showed slight decrease with 24 days of treatment. In contrast, for *KRAS* WT SW48 cells, the expression levels of EpCAM and EGFR kept stable during the treatment within 24 days; HER2 and HER3 expression showed significant decrease during the 24 days treatment. It should also be mentioned that despite fewer binding sites on the cells surface for anti‐EGFR SERS nanotags may be expected upon cetuximab treatment, the expression of EGFR detected by both SERS assay and flow cytometry showed no significant decrease for both SW480 (Figure 4B) and SW48 (Figure 5B) cells during treatment, which may be ascribed to the non‐saturating amount of cetuximab used in the assay, allowing for a consistent anti‐EGFR SERS binding before and after treatment.

The discrepancy of treatment response to cetuximab between *KRAS* mutant (SW480) and WT (SW48) cell lines is attributed to the following factors: (i) *KRAS* is the downstream effector of EGFR signalling pathway, thus, the genomic alterations (*KRAS* mutant) allow sustained MAPK signalling despite EGFR inhibition, leading to cell growth and proliferation.^[^ ^3^, ^7^, ^28^ ^]^ (ii) The increase in cell heterogeneity is common in the adaptive resistant group to treatment, which could be ascribed to the changes in gene expression or clonal selection of the resistant cells during treatment.^[^ ^9^, ^29^ ^]^ Aberrant activation by HER2 gene amplification is correlated with worse prognosis, and amplification of HER2 also leads to the failure response during anti‐EGFR therapy.^[^ ^19^, ^30^ ^]^ In this study, HER2 overexpression was observed in SW480 cells before treatment and no increase in expression was observed within 24 days treatment. It may be beneficial to examine HER2 expression over a longer time. (iii) It is interesting that in SW48 *KRAS* WT cells, treatment with anti‐EGFR caused a rapid reduction in HER2 and HER3 expression, while mean EGFR was unchanged. It has been demonstrated that dual HER2‐targeted treatment with trastuzumab and pertuzumab, could prevent the formation of heterodimers HER2−HER3,^[^ ^31^ ^]^ and our data suggest such a treatment may further boost the effectiveness of anti‐EGFR to reduce signalling through this axis. Moreover, our data raise the prospect to treat patients with HER2‐amplified CRC with anti‐EGRF drugs, to reduce HER2/3 expression.

## 3 CONCLUSIONS AND PERSPECTIVES

In summary, we used a SERS assay to measure the phenotypic evolution of four cell surface markers on *KRAS* mutant SW480 cells and *KRAS* wild‐type SW48 cells before and during anti‐EGFR treatment. Quantitation from SERS was in accordance with results from flow cytometry, which confirmed that SW48 cells (*KRAS* WT) are more sensitive to EGFR‐targeted inhibition, with a greater decrease of HER2 and HER3 expression levels during treatment, while EpCAM and EGFR expression remained stable. For *KRAS* mutant SW480 cells, HER2 expression slightly decreased, and HER3 expression increased within 24 days of treatment while the expression levels of EpCAM and EGFR remained stable throughout the experiment. Most importantly, the broadening of SERS signal distribution curves was observed for all the four biomarkers in SW480 cells in response to treatment, which is related to the tumor cell heterogeneity. Although it's still too early to confirm the prognostic marker because more tests in cell lines and clinical patient samples are required in the future work, the use of SERS has been demonstrated the great advantages in a simple (single laser excitation) and affordable laboratory system (assay time of 1 h) for characterizing multiple cell surface protein expression which is useful for investigating responses to drug treatments. We thus believe the SERS assay has the great potential for monitoring tumor evolution and therapeutic response non‐invasively in real‐time using blood samples. The future work on different patient cohorts will be conducted to demonstrate the translational pathway of the proposed SERS assay in clinical settings.

## 4 EXPERIMENTAL SECTION

### 4.1 Materials and reagents

4‐mercaptobenzoic acid (MBA), 7‐mercapto‐4‐methylcoumarin (MMC), and 5,5′‐dithiobis‐(2‐nitrobenzoic acid) (DTNB) were provided by Merck. 2,3,5,6‐tetrafluoro‐4‐mercaptobenzoic acid (TFMBA) was supplied by TCI (Japan). AuNPs were synthesized with HAuCl_4_·3H_2_O and sodium citrate tribasic dihydrate, both were ordered from Merck. IgG isotype control (monoclonal mouse IgG_1_, MAB002), anti‐EGFR monoclonal antibody (cetuximab biosimilar, recombinant monoclonal human IgG_1_ clone, MAB9577), anti‐EpCAM (monoclonal mouse IgG_2B_ Clone, MAB9601), anti‐HER2 (monoclonal mouse IgG_2B_ clone, MAB1129), and anti‐HER3 (monoclonal mouse IgG_1_ clone, MAB3481) were provided by R&D systems (Australia). The same antibodies were used for both SERS and flow cytometry. 3,3′‐dithiobis(sulfosuccinimidyl propionate) (DTSSP) were provided by Merck for conjugation of antibodies with AuNPs. The secondary antibody Alexa Fluor 488 goat anti‐mouse IgG antibody was ordered from Life Technologies (A‐11001, Australia), human IgG APC‐conjugated antibody (F0135) was supplied by R&D systems. Cell line SW480 and SW48 were supplied by ATCC.

### 4.2 Apparatus

The size and shape of AuNPs were observed with transmission electron microscope (Philips CM10). The hydrodynamic size and zeta potential of AuNPs were determined by Zetasizer (Malvern, UK). The optical characteristics of AuNPs were examined with UV–vis spectroscopy (Cary 5000, Agilent, USA) to investigate the optical properties of AuNPs. The shaker Intelli‐Mixer (RM‐2M, ELMI Ltd, Riga, Latvia) and ThermoMixer C (Eppendorf, Hamburg, Germany) were used for incubation purposes. SERS measurements were performed with the Raman microscope (IM‐52, Snowy Range Instruments) with a 785‐nm wavelength laser for excitation. Flow cytometry was tested by BD LSRFortessa X‐20 flow cytometer.

### 4.3 Gold nanoparticles synthesis

AuNPs were made as reported,^[^ ^32^ ^]^ where gold chloride was reduced with sodium citrate. In brief, HAuCl_4_ (0.01% w/v, 50 ml) was heated until boiling point followed by addition of sodium citrate (1% w/v, 0.35 ml). The solution was kept boiling with stirring for 15 min, which generates 60 nm AuNPs in diameter.

### 4.4 SERS nanotags preparation

AuNPs were functionalized with Raman molecules and antibodies to prepare Ab‐SERS nanotags, as previously reported.^[^ ^33^ ^]^ To be brief, 1.5 ml of the as‐prepared AuNPs colloidal was concentrated into 1.0 ml, followed by the addition of 10 µl of 1 mM ethanolic solution of Raman reporter. The mixture was then incubated at room temperature (RT) under shaking (60 rpm, Mode 45, Intelli‐Mixer) for 5 h, which will ensure the form of self‐assembled monolayer (SAM) on AuNPs surface. Subsequently, the residual reactants were removed by centrifugation of the mixture at 5500 rpm for 8 min. The mixture was then re‐dispersed in PBS (0.1 mM, 300 µl) to reach with DTSSP‐linked antibody, which was prepared by mixing 5 µl of 1 mg/ml DTSSP (in 5 mM sodium citrate buffer, pH 5.3) and 10 µl of 0.5 mg/ml antibody (in PBS) under shaking (300 rpm, ThermoMixer C, Eppendorf) for 30 min. The mixture was then incubated under shaking (300 rpm) at RT for 30 min and kept at 4°C overnight. After that, the mixture was centrifuged at 4°C using the speed of 3300 rpm for 10 min to remove the excess antibodies. To block the non‐specific binding sites on surface of nanotags, SERS nanotags were re‐dispersed in 300 µl of BSA solution (0.05% w/v in PBS) under 350 rpm shaking for 30 min at RT.

### 4.5 Cell surface proteins profiling

The two cell lines SW480 and SW48 were validated by short‐tandem repeat profiling. Specifically, SW480 cells were cultured using RPMI 1640 medium with 10% v/v fetal bovine serum (FBS) and a mix of the antibiotics (penicillin of 100 IU/ml, and streptomycin of 100 µg/ml) at 37°C, 5% CO_2_. The existing gene of S480 cell was not disrupted or knocked out, thus the KRAS mutant in SW480 cell was in its active state. Instead, SW48 cells were cultured using L­15 Medium with FBS (10% v/v) and a mix of the antibiotics under 37°C but without gas exchange with atmospheric air.

To profile the cell surface markers after treatment with cetuximab, cells (5 × 10^5^ cells/flask in 5 ml cell culture media) were seeded in a T25 flask (Greiner Bio‐One, Austria). The cells were then treated with 1 µg/ml of cetuximab for 3, 7, 10, 17 and 24 days. For the longer treatment time (7, 10, 17 and 24 day), cells were passaged every 3 or 4 days, in which 5 × 10^5^ cetuximab‐treated cells were planted in 5 ml cell culture media with 1 µg/ml of cetuximab. The cells were then collected, washed and re‐dispersed in the PBS buffer (with 1% v/v FBS) to get 5 × 10^5^ cells/ml of cells suspension for the profiling of cell surface proteins with SERS nanotags and flow cytometry, both assays were conducted with live cells.

The method for labelling cells with Ab‐SERS nanotags has been reported.^[^ ^9^ ^]^ The four Raman reporters MBA, DTNB, MMC, and TFMBA were adopted for functionalizing AuNPs and detecting EpCAM, EGFR, HER2, and HER3, respectively. The Ab‐SERS nanotags has variable signal enhancement, which is ascribed to the different cross section of Raman scattering for the Raman reporter MBA, DTNB, MMC and TFMBA.^[^ ^34^ ^]^ IgG‐conjugated AuNPs (IgG‐SERS nanotags) were used as an internal negative control, which will minimize the variation of SERS signals when multiple nanotags are present. Cells suspension (5 × 10^5^ cells/ml, 200 µl) were incubated with the four Ab‐SERS nanotags mixes (30 µl each) under 300 rpm shaking for 30 min at 37°C, followed by being centrifuged (2000 rpm, 1 min) and washed with buffer (PBS with 1% v/v of FBS) for 4 times, where 200 µl of buffer was used for each wash. Subsequently, the labelled cells were re‐dispersed in 60 µl of buffer for SERS reading. SERS spectra were acquired with 785‐nm excitation laser by the Raman Microscope under incident laser power of 70 mW and integration of 1 s. For each sample of labelled cells, 150 SERS spectra were continuously acquired with Raman spectroscopy to reflect different portion of cells in the solution that were undergoing Brownian movement. The assay time including cell collection, cell labelling with Ab‐SERS nanotags and Raman detection was about 1 h. It should also be mentioned that tumor cells in the clinical samples could be identified using similar approach of the proposed assay, however, the blood sample needs to be pre‐treated to remove the red blood cells and leucocytes.^[^ ^9^ ^]^ For each SERS spectrum (Figure 1B), SERS signal intensities of the characteristic peaks represent the expression levels of the respective biomarkers (1078, 1180, 1340 and 1380 cm^−1^ for EpCAM, HER2, EGFR and HER3, respectively). The SERS frequency vs. intensity curve was generated by counting the frequency of Raman intensity from 150 measurements (i.e., 150 intensity data at 1078 cm^−1^ for EpCAM, the frequency of each intensity interval (0–50, 50–100, …) were counted, respectively). 150 SERS spectra were also recorded for each sample of cells labelled with four Raman reporter‐IgG SERS nanotags. The SERS frequency vs. intensity at 1078, 1180, 1340 and 1380 cm^−1^ were also plotted when the IgG‐SERS nanotags were used.

For flow cytometry, cells (2 × 10^6^/ml, 100 µl) re‐suspended in FACS buffer (PBS with 3% v/v FBS, 0.5% w/v BSA and 1 mM EDTA) were mixed with antibody (0.5 µg/µl, 5 µl of either anti‐EpCAM, anti‐EGFR, anti‐HER2, or anti‐HER3 antibody or IgG) at RT for 30 min under gentle rotation (60 rpm, Mode 03, Intelli‐Mixer) to avoid the sedimentation of cells and facilitate receptor and antibody interaction. After being washed 2 times with FACS buffer (200 µl for each wash), the cells labelled with anti‐EpCAM, anti‐HER2 or anti‐HER3 antibody were dispersed in 200 µl Alexa Fluor 488 goat anti‐mouse IgG antibody (1 µg/ml) and incubated at RT for 30 min under gentle rotation (60 rpm, Mode 03, Intelli‐Mixer). The cells labelled with anti‐EGFR antibody were redispersed in FACS buffer (200 µl) and then 10 µl of secondary antibody (human IgG APC‐conjugated antibody) was added for 30 min under gentle rotation (60 rpm, Mode 03, Intelli‐Mixer). After being washed with FACS buffer to discard the excess secondary antibody, the cells were resuspended in FACS buffer (500 µl) supplemented with 5 µl of DAPI solution (0.5 µg/ml) for flow cytometry. The experiments were performed with three technical replicates. It should be noted that the different sources of primary antibody may generate the curves of IgG control slightly different in flow cytometry. The percentage of cells expressing the corresponding biomarker is presented by using isotype‐matched IgG as control, and the gate is set where the “expression” for IgG control should be less than 1.0%.^[^ ^35^ ^]^ The assay time including cell collection, cell labelling with primary and secondary antibodies, and detection with flow cytometer was about 1.7 h. The expression of each biomarker could also be presented by the ratio of MFI for stained and control samples.

## CONFLICT OF INTEREST

There is no conflict of interest to declare.

## AUTHOR CONTRIBUTIONS

Nana Lyu, Mark P. Molloy and Yuling Wang designed the research, analysed the results, and wrote the manuscript. Bernadette Pedersen and Elena Shklovskaya involved in the sample preparation, detection and data analysis by flow cytometry. Helen Rizos, Mark P. Molloy and Yuling Wang commented on the draft.

## Supporting information




**Supporting Information**: The supplementary materials are available online. Figure S1: Hydrodynamic size distribution (by number) of AuNPs before and after conjugation with Raman reporter molecule and antibody measured by DLS; Table S1: Hydrodynamic size of AuNPs before and after functionalization measured by DLS and zeta potential measured by ELS; Figure S2: Surface marker expression profiles for SW48 cells before drug treatment (0 day) measured by SERS assay and flow cytometry; Figures S3–S7: Cell surface expression in SW480 cells upon cetuximab treatment for 3, 7, 10, 17 and 24 day measured by SERS assay and flow cytometry; Figure S8: The histogram overlay of surface marker expression profiles for SW480 cells measured by SERS assay over a 24‐day time course of cetuximab treatment; Figure S9: The histogram overlay of surface marker expression profiles for SW480 cells measured by flow cytometry over a 24‐day time course of cetuximab treatment; Figures S10–S14: Cell surface expression in SW48 cells upon cetuximab treatment for 3, 7, 10, 17 and 24 day measured by SERS assay and flow cytometry; Figure S15: The histogram overlay of surface marker expression profiles for SW48 cells measured by SERS assay over a 24‐day time course of cetuximab treatment; Figure S16: The histogram overlay of surface marker expression profiles for SW48 cells measured by flow cytometry over a 24‐day time course of cetuximab treatment.
