# Supplementary material for: SERS characterization of colorectal cancer cell surface markers upon anti‐EGFR treatment
Source: Exploration (Beijing). 2022 May 9;2(3):20210176. doi: 10.1002/EXP.20210176 (PMC10190927; doi:10.1002/EXP.20210176)
Supplement: Supplementary file 1 — Supporting Information: The supplementary materials are available online. Figure S1: Hydrodynamic size distribution (by number) of AuNPs before and after conjugation with Raman reporter molecule and antibody measured by DLS; Table S1: Hydrodynamic size of AuNPs before and after functionalization measured by DLS and zeta potential measured by ELS; Figure S2: Surface marker expression profiles for SW48 cells before drug treatment (0 day) measured by SERS assay and flow cytometry; Figures S3–S7: Cell surface expression in SW480 cells upon cetuximab treatment for 3, 7, 10, 17 and 24 day measured by SERS assay and flow cytometry; Figure S8: The histogram overlay of surface marker expression profiles for SW480 cells measured by SERS assay over a 24‐day time course of cetuximab treatment; Figure S9: The histogram overlay of surface marker expression profiles for SW480 cells measured by flow cytometry over a 24‐day time course of cetuximab treatment; Figures S10–S14: Cell surface expression in SW48 cells upon cetuximab treatment for 3, 7, 10, 17 and 24 day measured by SERS assay and flow cytometry; Figure S15: The histogram overlay of surface marker expression profiles for SW48 cells measured by SERS assay over a 24‐day time course of cetuximab treatment; Figure S16: The histogram overlay of surface marker expression profiles for SW48 cells measured by flow cytometry over a 24‐day time course of cetuximab treatment. [file EXP2-2-20210176-s001.DOCX]

***Supplementary Material***

**SERS characterization of colorectal cancer cell surface markers upon anti-EGFR treatment**

Nana Lyu^1^, Bernadette Pedersen^2^, Elena Shklovskaya^2^, Helen Rizos^2^, Mark P. Molloy^3, 🖂^ and Yuling Wang^1, 🖂^

1. ARC Center of Excellence for Nanoscale BioPhotonics and School of Natural Sciences, Faculty of Science and Engineering, Macquarie University, Sydney, NSW 2109, Australia

2. Department of Biomedical Sciences, Faculty of Medicine, Health and Human Sciences, Macquarie University, Sydney, NSW 2109, Australia

3. Bowel Cancer and Biomarker Laboratory, School of Medical Sciences, Kolling Institute, The University of Sydney, NSW 2006, Australia

**^🖂^** Corresponding author: [m.molloy@sydney.edu.au](mailto:m.molloy@sydney.edu.au); [yuling.wang@mq.edu.au](mailto:yuling.wang@mq.edu.au)


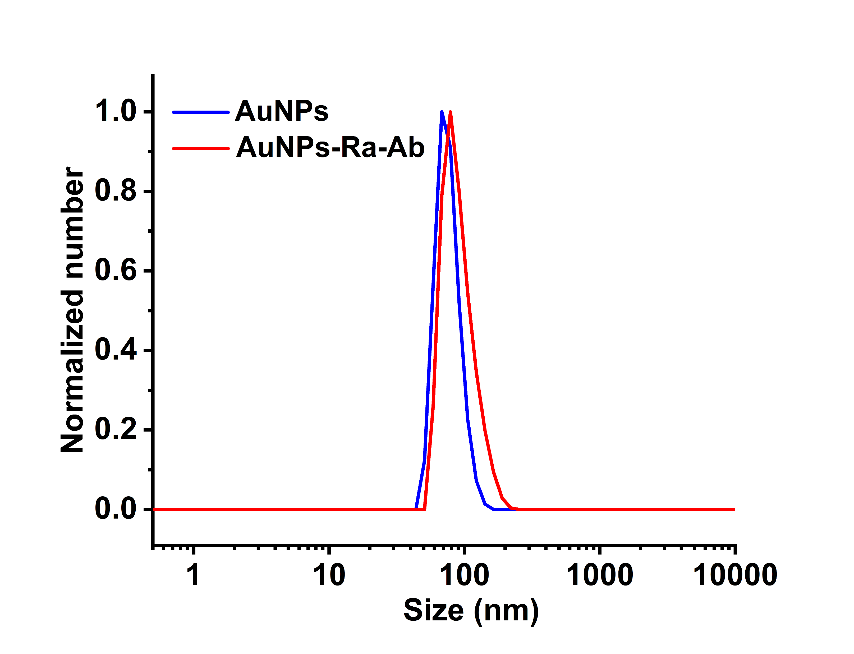


**Figure S1.** Hydrodynamic size distribution (by number) of AuNPs before and after conjugation with Raman reporter molecule (Ra, 5,5’-dithiobis-(2-nitrobenzoic acid) (DTNB)) and antibody (Ab, anti-EGFR) measured by dynamic light scattering (DLS).

**Table S1.** Hydrodynamic size of AuNPs before and after functionalization measured by DLS and zeta potential measured by electrophoretic light scattering (ELS) (n=3)

| Sample | Size (nm) | Zeta potential (mV) |
| --- | --- | --- |
| AuNPs | 68.1 ± 1.4 | -23.4 ± 1.8 |
| AuNPs-Ra-Ab | 78.8 ± 3.5 | -20.1 ± 1.6 |


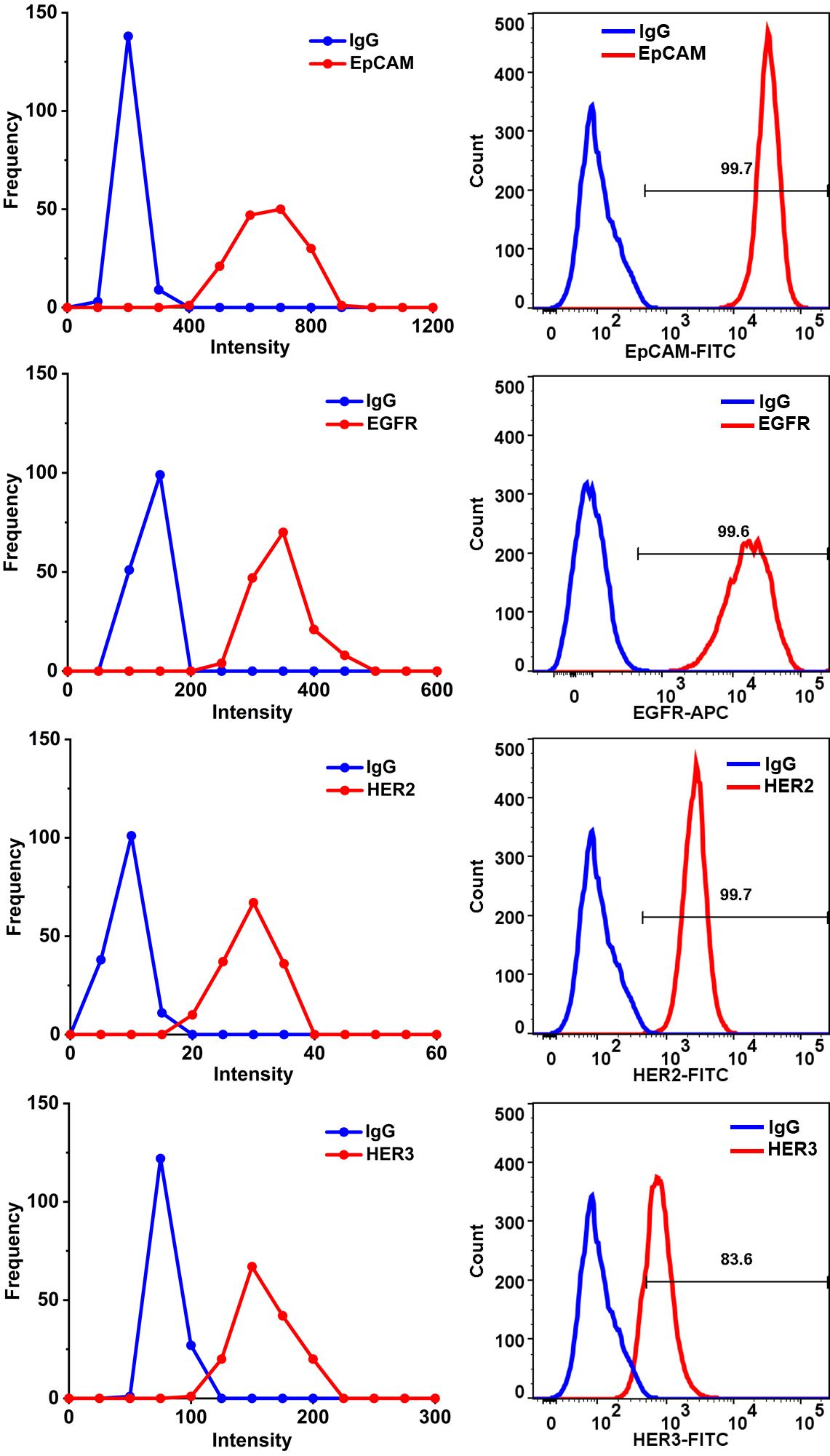


**Figure S2.** Surface marker expression profiles for SW48 cells before drug treatment (0 day). (Left) SERS assay of the distribution of four markers (EpCAM, EGFR, HER2 and HER3) on cells’ surface; (Right) Flow cytometry analysis of cell surface marker expression.


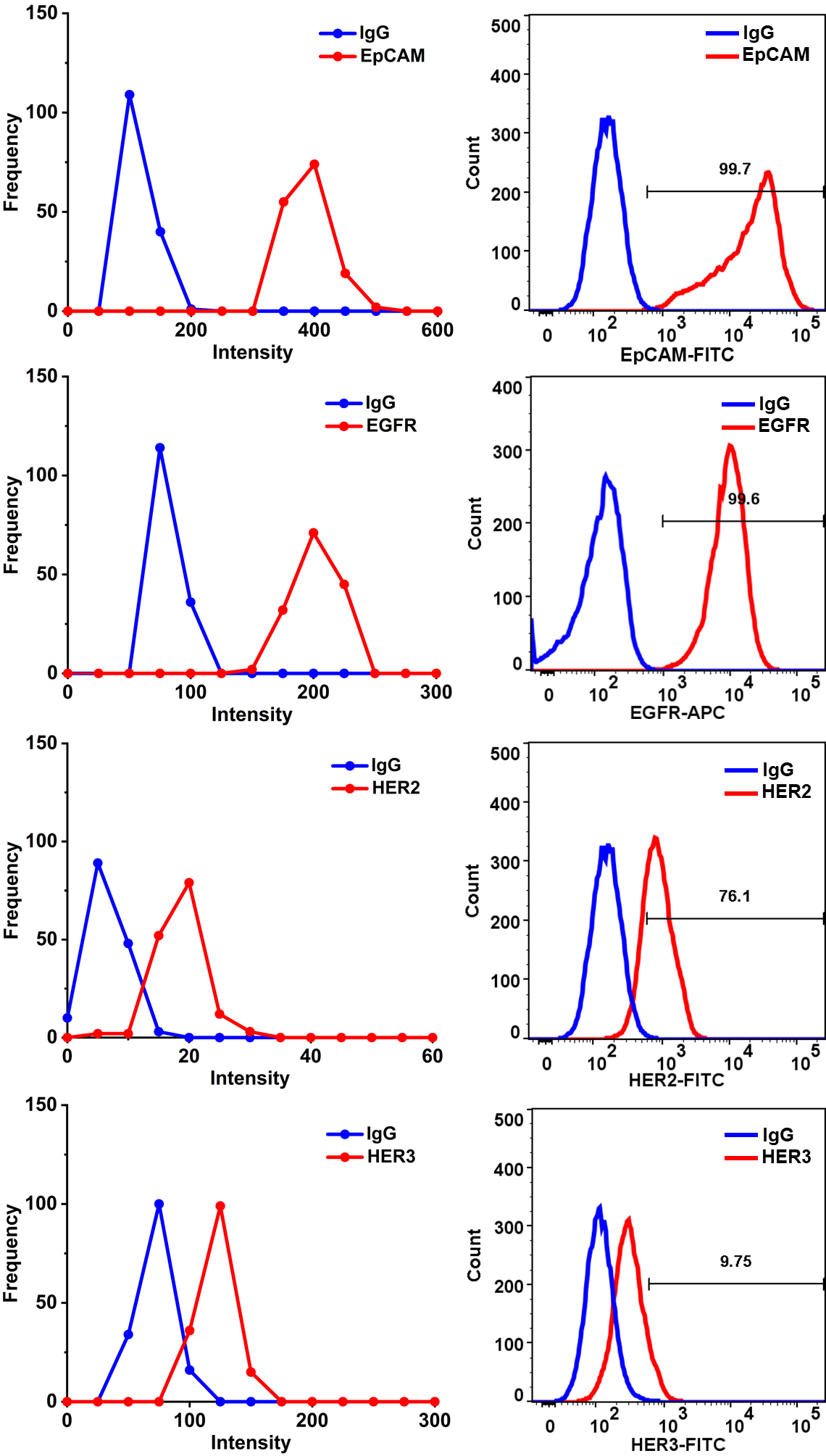


**Figure S3.** Surface marker expression profiles for SW480 cells after treatment with cetuximab for 3 days. (Left) SERS assay of the distribution of four markers (EpCAM, EGFR, HER2 and HER3) on cells’ surface; (Right) Flow cytometry analysis of cell surface marker expression.

**
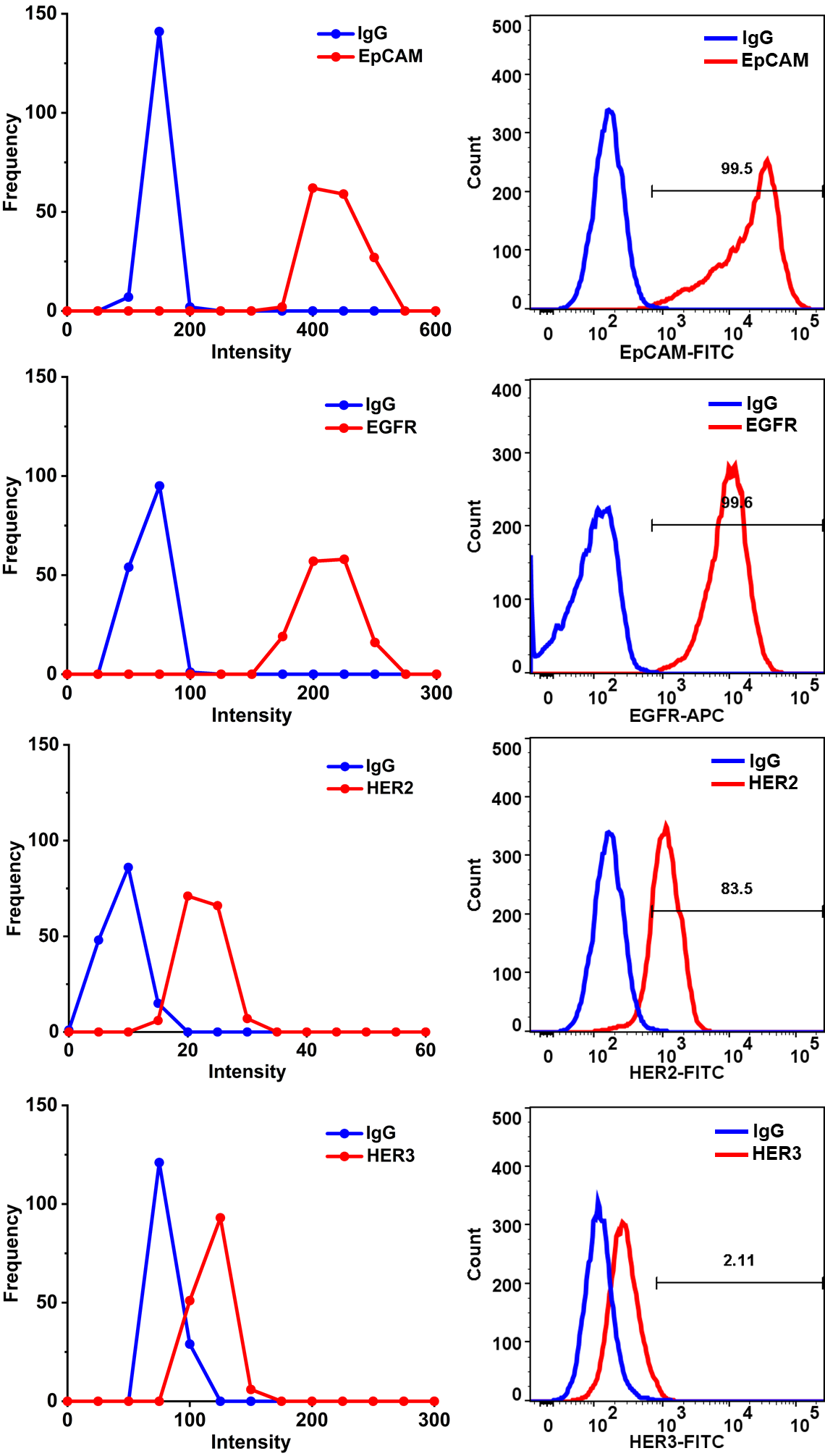
**

**Figure S4.** Surface marker expression profiles for SW480 cells after treatment with cetuximab for 7 days. (Left) SERS assay of the distribution of four markers (EpCAM, EGFR, HER2 and HER3) on cells’ surface; (Right) Flow cytometry analysis of cell surface marker expression.

**
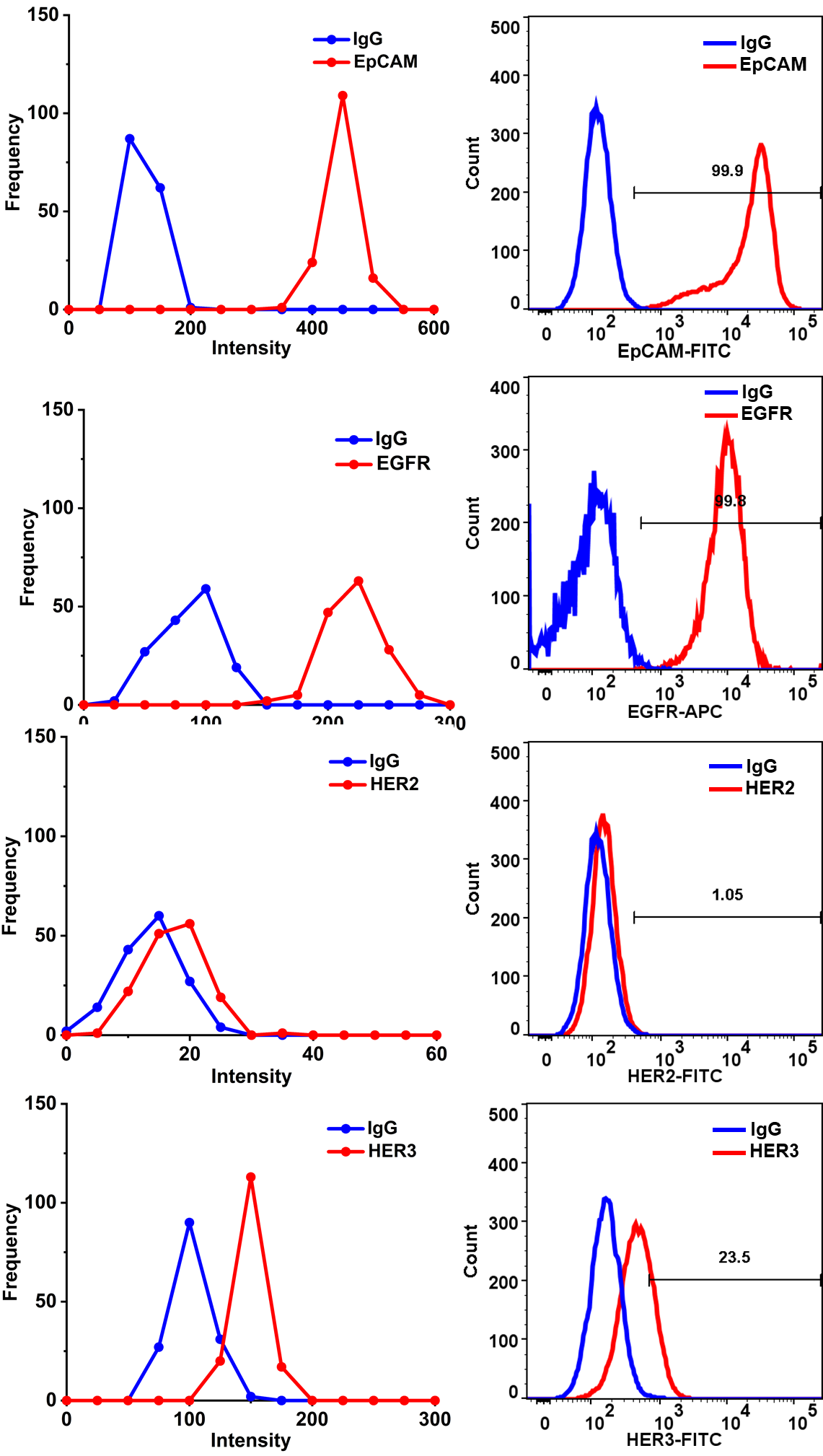
**

**Figure S5.** Surface marker expression profiles for SW480 cells after treatment with cetuximab for 10 days. (Left) SERS assay of the distribution of four markers (EpCAM, EGFR, HER2 and HER3) on cells’ surface; (Right) Flow cytometry analysis of cell surface marker expression.


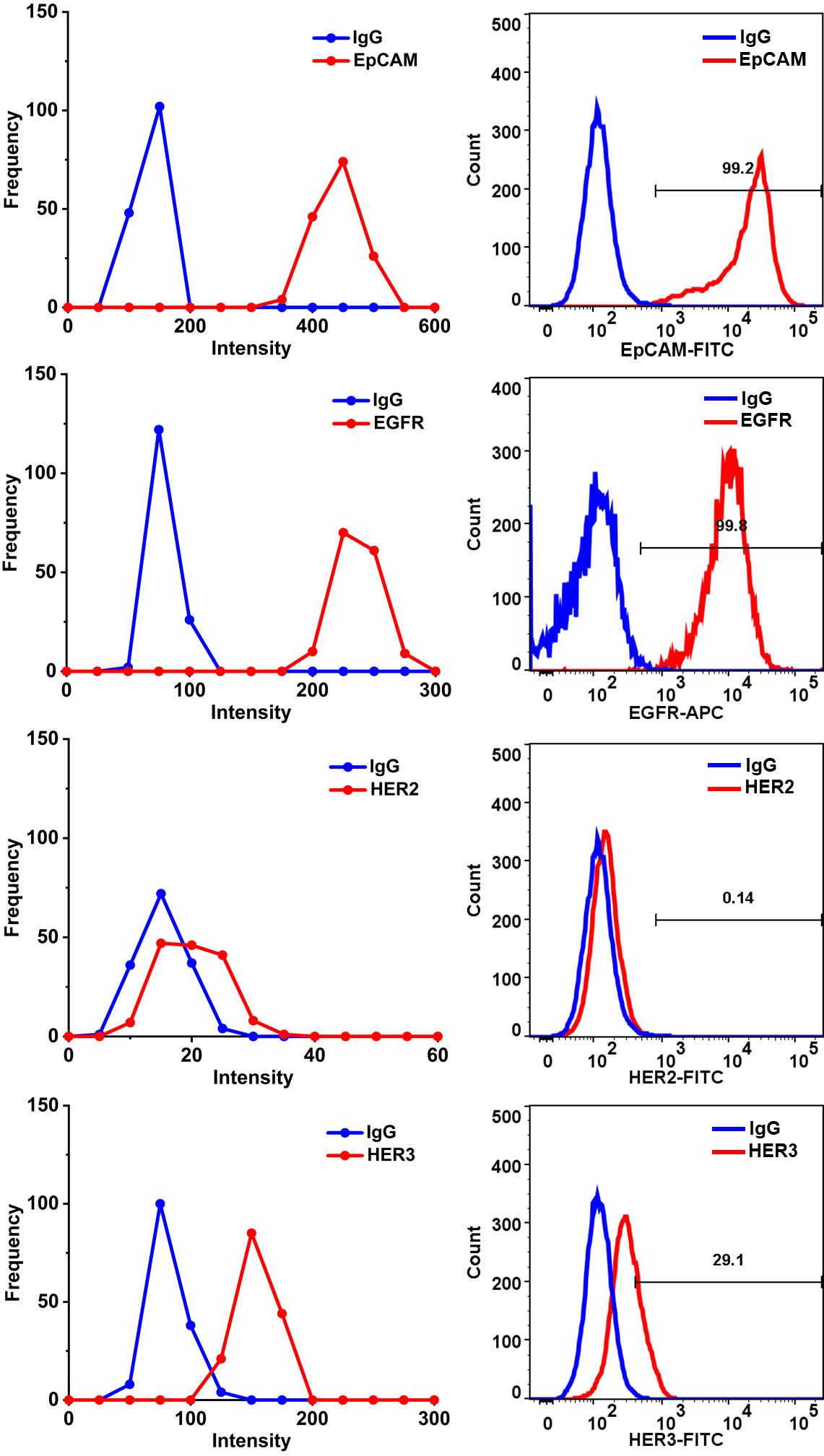


**Figure S6.** Surface marker expression profiles for SW480 cells after treatment with cetuximab for 17 days. (Left) SERS assay of the distribution of four markers (EpCAM, EGFR, HER2 and HER3) on cells’ surface; (Right) Flow cytometry analysis of cell surface marker expression.

**
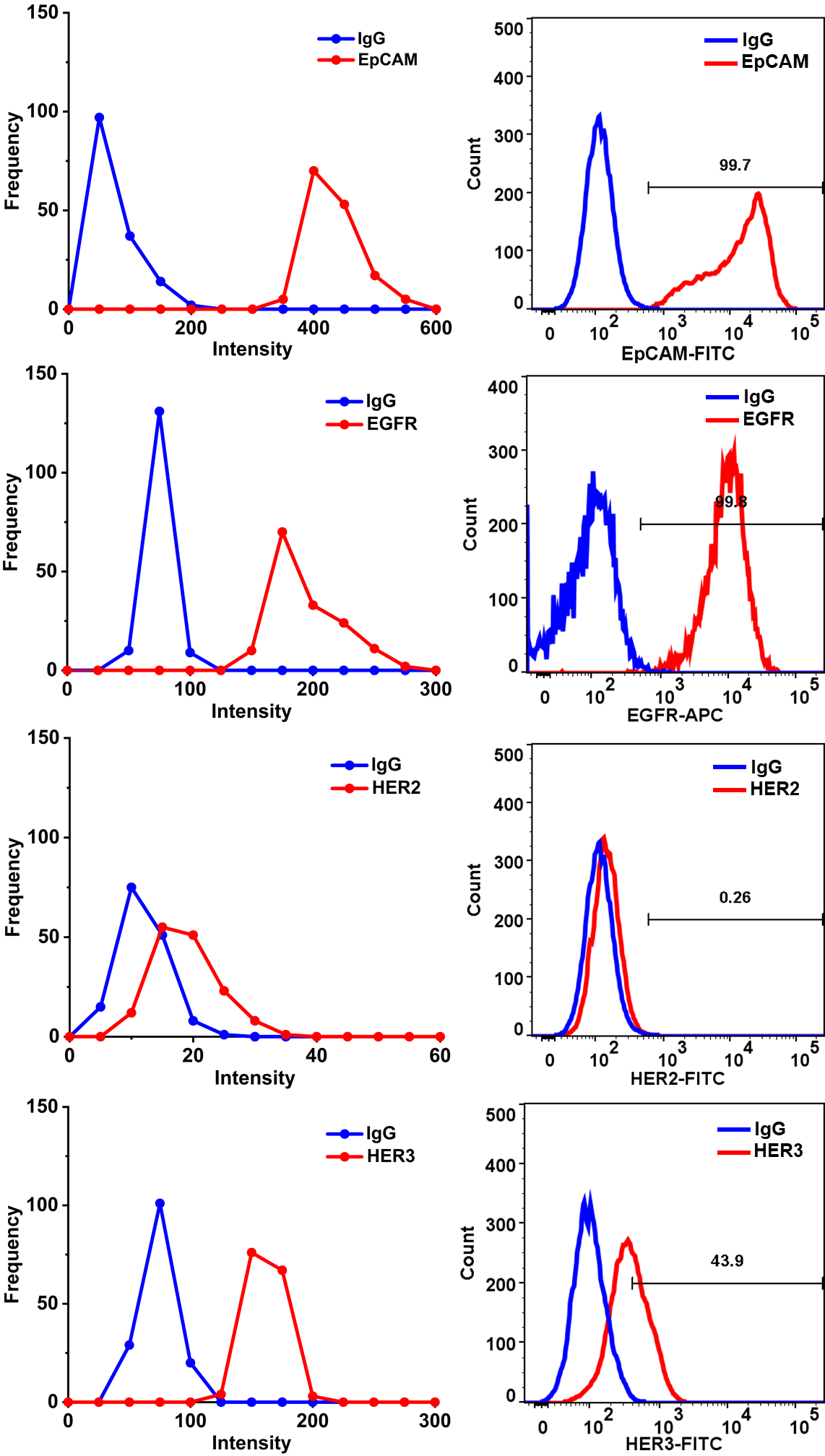
**

**Figure S7.** Surface marker expression profiles for SW480 cells after treatment with cetuximab for 24 days. (Left) SERS assay of the distribution of four markers (EpCAM, EGFR, HER2 and HER3) on cells’ surface; (Right) Flow cytometry analysis of cell surface marker expression.


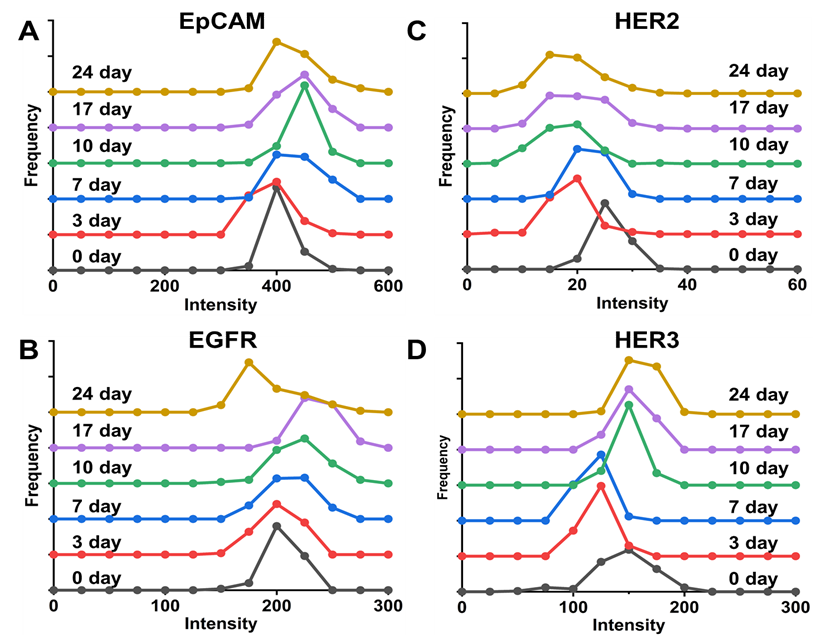


**Figure S8.** SERS assay of surface marker expression profiles for SW480 cells before drug treatment (0 day) and upon cetuximab treatment for 3, 7, 10, 17 and 24 days.


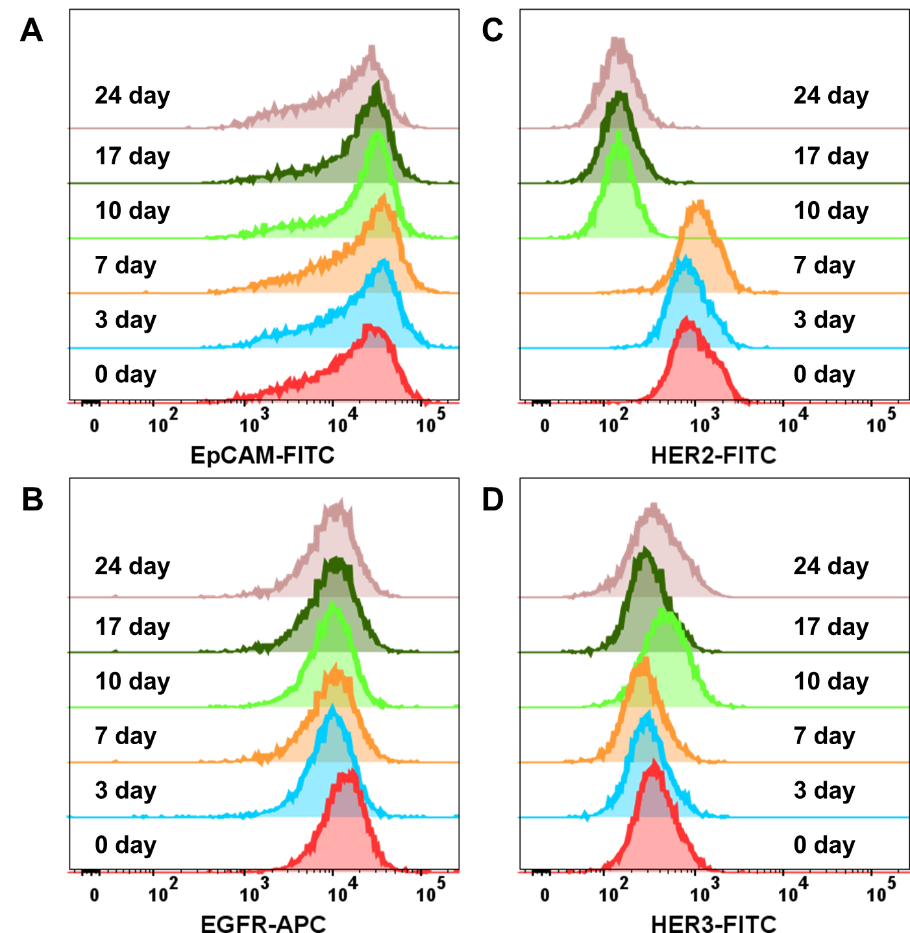


**Figure S9.** Flow cytometry analysis of surface marker expression profiles for SW480 cells before drug treatment (0 day) and upon cetuximab treatment for 3, 7, 10, 17 and 24 days.

**
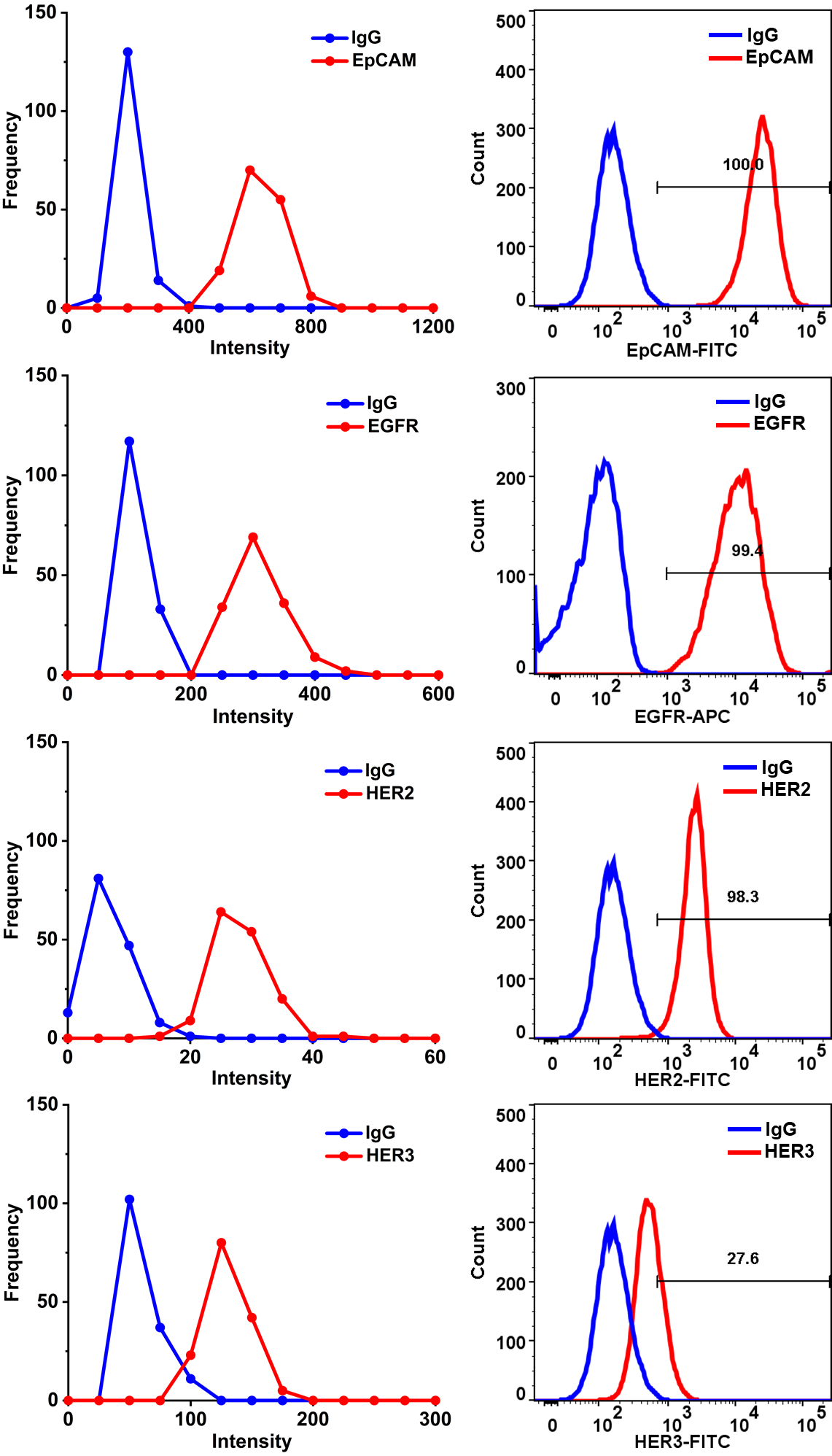
**

**Figure S10.** Surface marker expression profiles for SW48 cells after treatment with cetuximab for 3 days. (Left) SERS assay of the distribution of four markers (EpCAM, EGFR, HER2 and HER3) on cells’ surface; (Right) Flow cytometry analysis of cell surface marker expression.

**
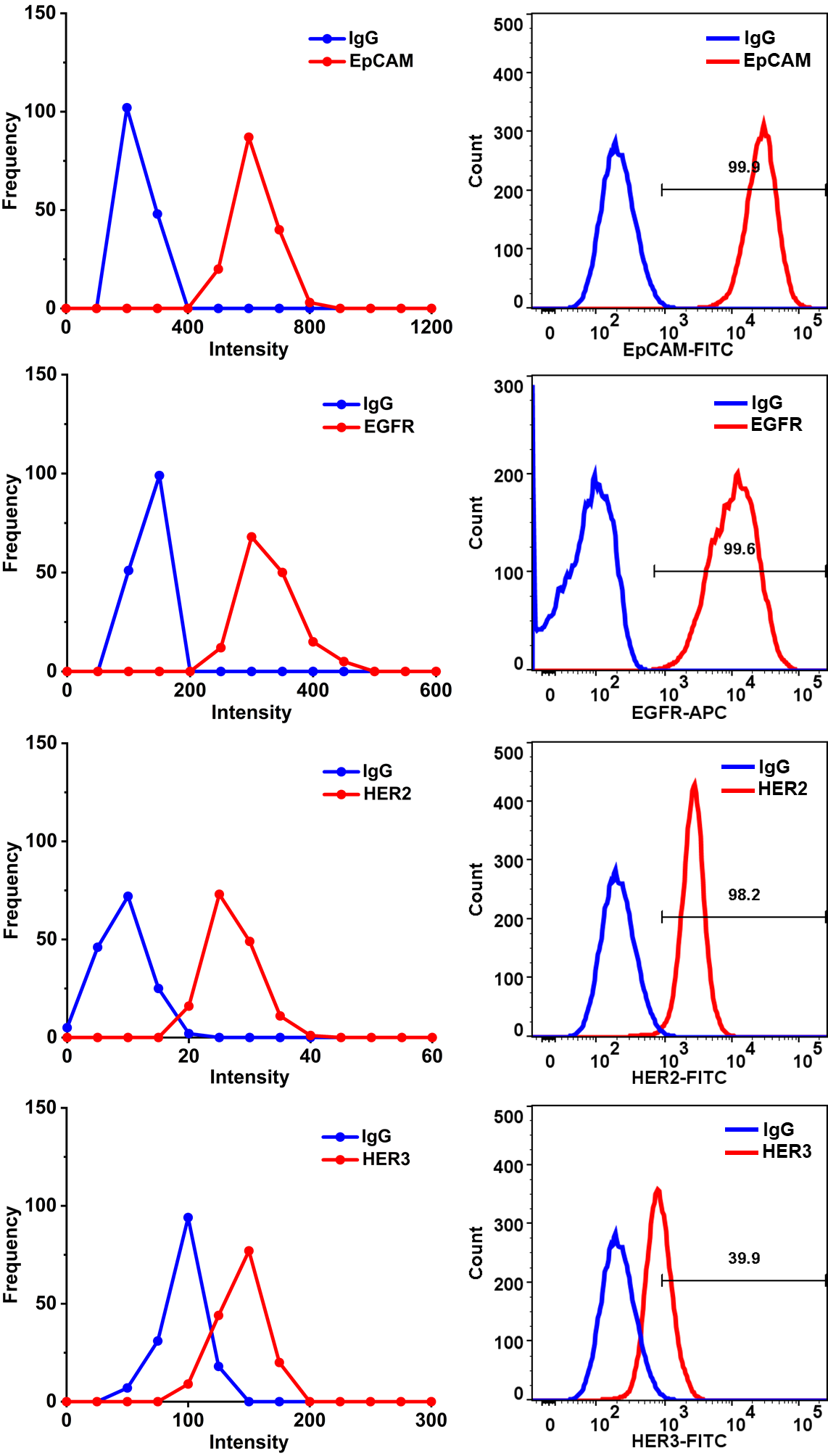
**

**Figure S11.** Surface marker expression profiles for SW48 cells after treatment with cetuximab for 7 days. (Left) SERS assay of the distribution of four markers (EpCAM, EGFR, HER2 and HER3) on cells’ surface; (Right) Flow cytometry analysis of cell surface marker expression.

**
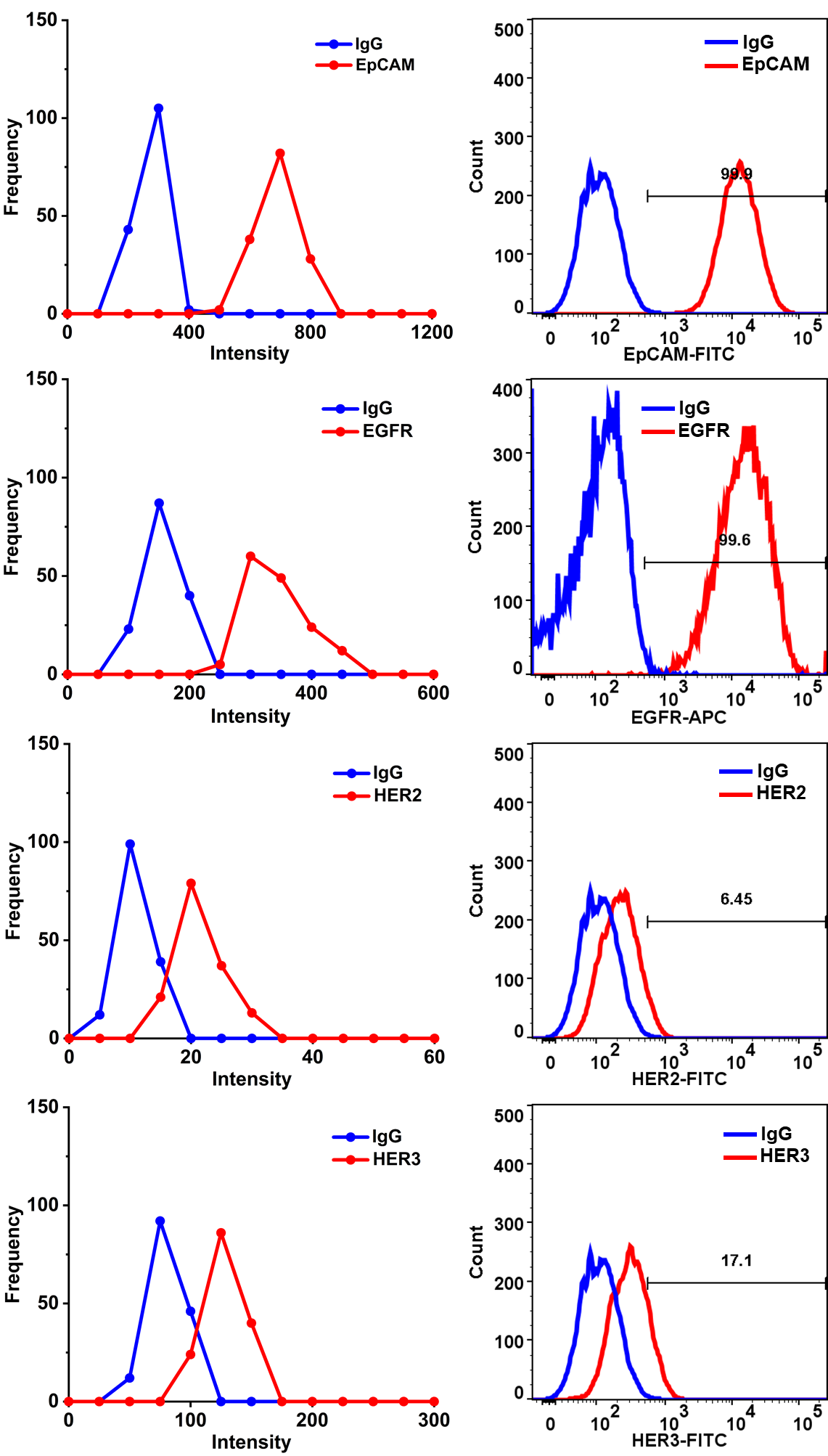
**

**Figure S12.** Surface marker expression profiles for SW48 cells after treatment with cetuximab for 10 days. (Left) SERS assay of the distribution of four markers (EpCAM, EGFR, HER2 and HER3) on cells’ surface; (Right) Flow cytometry analysis of cell surface marker expression.

**
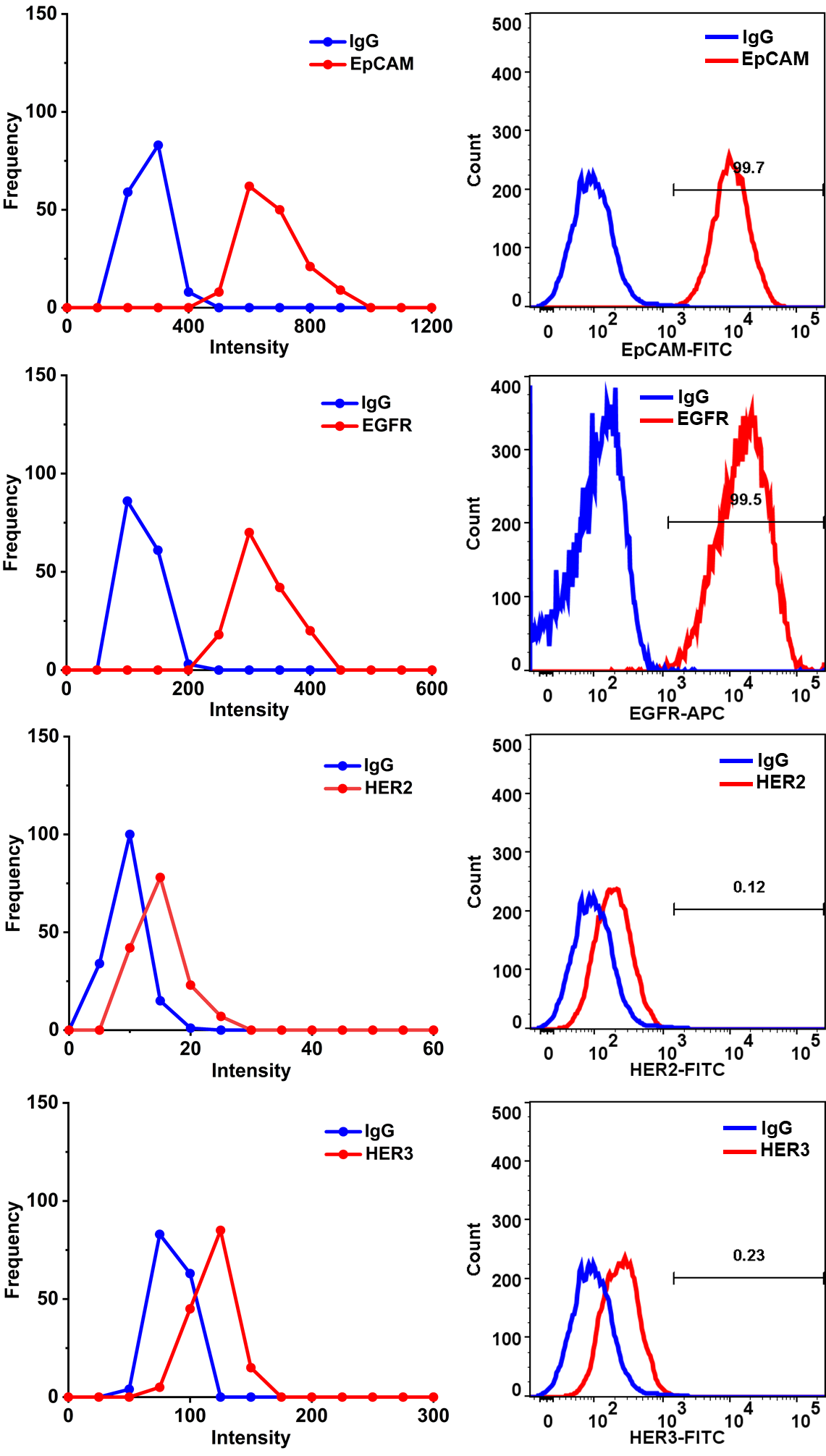
**

**Figure S13.** Surface marker expression profiles for SW48 cells after treatment with cetuximab for 17 days. (Left) SERS assay of the distribution of four markers (EpCAM, EGFR, HER2 and HER3) on cells’ surface; (Right) Flow cytometry analysis of cell surface marker expression.

**
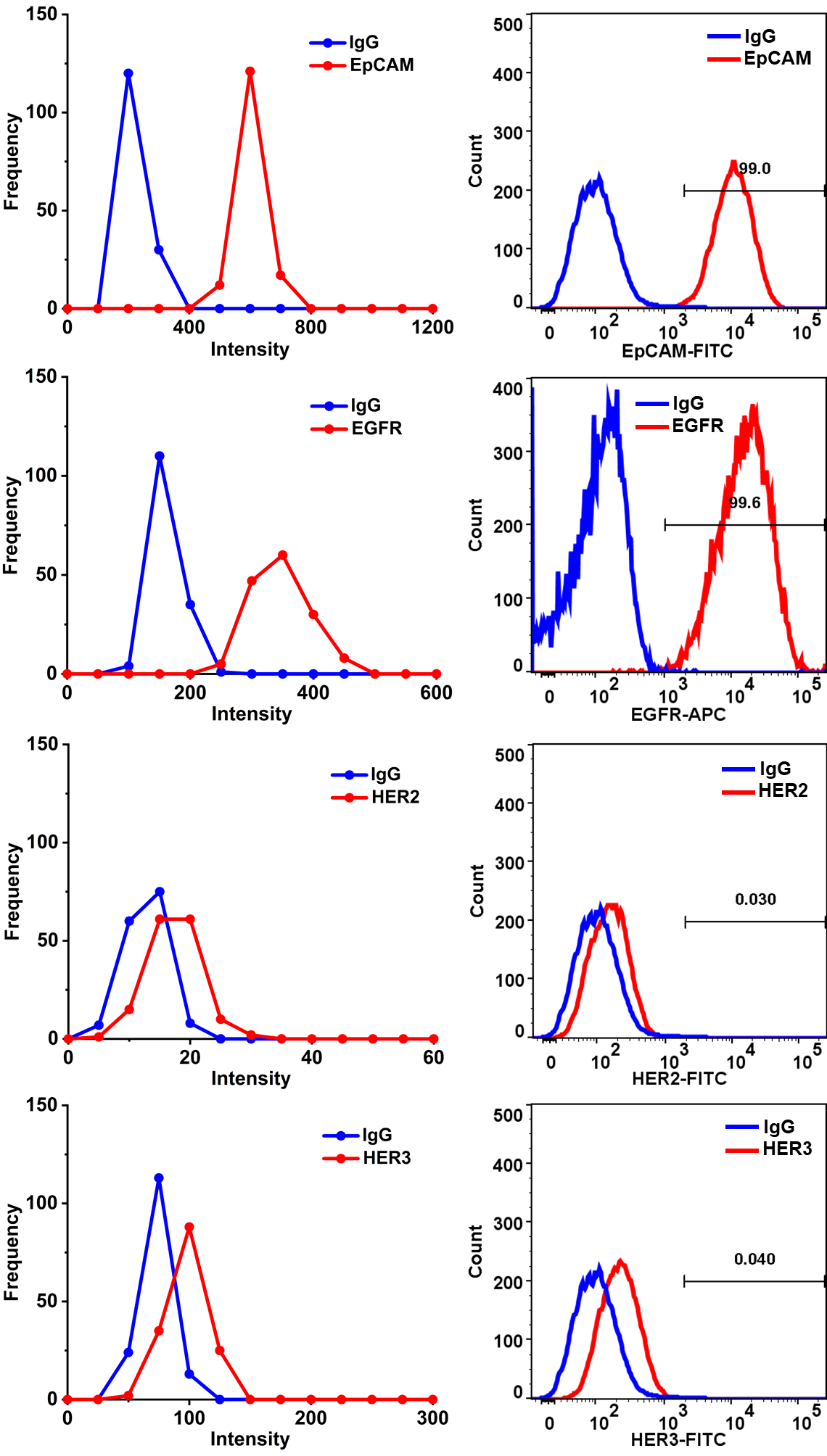
**

**Figure S14.** Surface marker expression profiles for SW48 cells after treatment with cetuximab for 24 days. (Left) SERS assay of the distribution of four markers (EpCAM, EGFR, HER2 and HER3) on cells’ surface; (Right) Flow cytometry analysis of cell surface marker expression.

**
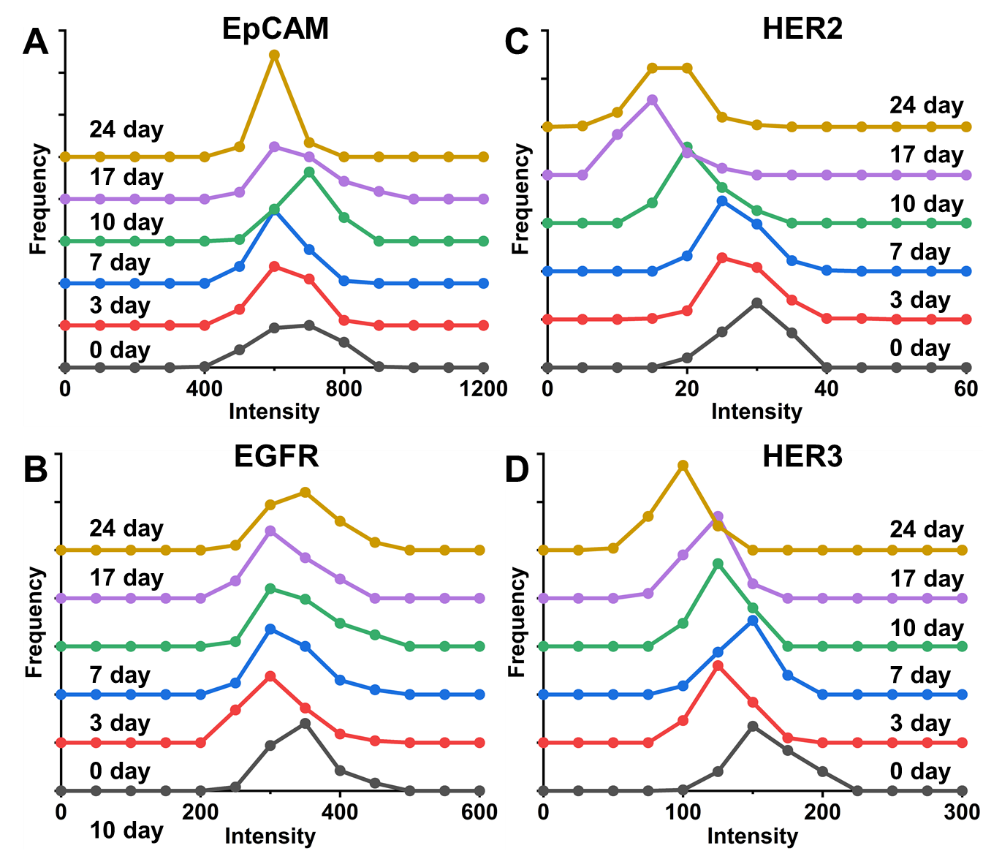
**

**Figure S15.** SERS assay of evolution of surface marker expression profiles for SW48 cells before drug treatment (0 day) and upon cetuximab treatment for 3, 7, 10, 17 and 24 days.


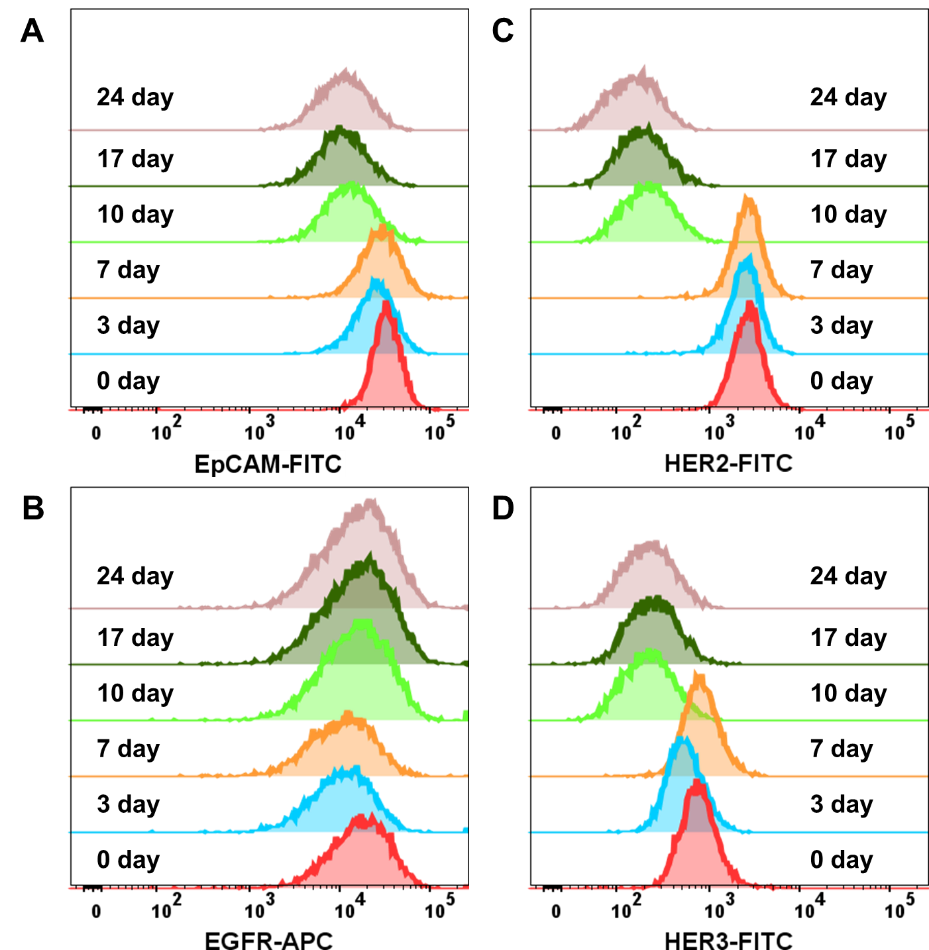


**Figure S16.** Flow cytometry assay of evolution of surface marker expression profiles for SW48 cells before drug treatment (0 day) and upon cetuximab treatment for 3, 7, 10, 17 and 24 days.
